# *Haemophilus parainfluenzae* expresses diverse lipopolysaccharide O-antigens using ABC transporter and Wzy polymerase-dependent mechanisms

**DOI:** 10.1016/j.ijmm.2013.08.006

**Published:** 2013-12

**Authors:** Rosanna E.B. Young, Brigitte Twelkmeyer, Varvara Vitiazeva, Peter M. Power, Elke K.H. Schweda, Derek W. Hood

**Affiliations:** a Department of Structural and Molecular Biology, University College London, Darwin Building, Gower Street, London WC1E 6BT, United Kingdom; b Department of Paediatrics, University of Oxford, United Kingdom^1^; c Clinical Research Centre, Karolinska Institutet, Novum, S-141 86 Huddinge, Stockholm, Sweden; d Division of Chemistry, IFM, Linköping University, SE-581 83 Linköping, Sweden; e MRC Harwell, Oxfordshire OX11 0RD, United Kingdom

**Keywords:** Lipopolysaccharide, O-antigen, Host–bacterial interaction, Cell surface, Pasteurellaceae

## Abstract

Lipopolysaccharide O-antigens are the basis of serotyping schemes for Gram negative bacteria and help to determine the nature of host–bacterial interactions. *Haemophilus parainfluenzae* is a normal commensal of humans but is also an occasional pathogen. The prevalence, diversity and biosynthesis of O-antigens were investigated in this species for the first time. 18/18 commensal *H. parainfluenzae* isolates contain a O-antigen biosynthesis gene cluster flanked by *glnA* and *pepB*, the same position as the *hmg* locus for tetrasaccharide biosynthesis in *Haemophilus influenzae*. The O-antigen loci show diverse restriction digest patterns but fall into two main groups: (1) those encoding enzymes for the synthesis and transfer of FucNAc4N in addition to the Wzy-dependent mechanism of O-antigen synthesis and transport and (2) those encoding galactofuranose synthesis/transfer enzymes and an ABC transporter. The other glycosyltransferase genes differ between isolates. Three *H. parainfluenzae* isolates fell outside these groups and are predicted to synthesise O-antigens containing ribitol phosphate or deoxytalose. Isolates using the ABC transporter system encode a putative O-antigen ligase, required for the synthesis of O-antigen-containing LPS glycoforms, at a separate genomic location. The presence of an O-antigen contributes significantly to *H. parainfluenzae* resistance to the killing effect of human serum in vitro. The discovery of O-antigens in *H. parainfluenzae* is striking, as its close relative *H. influenzae* lacks this cell surface component.

## Introduction

The O-antigen (OAg) component of cell surface lipopolysaccharide (LPS) is one of the most diverse structures found in Gram negative bacteria, differing both within and between species. It is the basis of typing schemes for many bacterial species, using antisera raised specifically against each OAg structure to test for reactivity. In some cases a correlation can be seen between OAg serotype and clinical symptoms due to the numerous roles that OAg plays in the modulation of bacterial–host interactions. For *Escherichia coli*, over 170 different OAg structures have been identified (Lundborg et al., 2010): each contains one to seven sugars per repeat unit (O-unit), with extra variation added by different sugar conformations, linkages, branching patterns and modifications. Other species are more conservative; for example, there are only seven known serotypes for *Aggregatibacter actinomycetemcomitans* (Kaplan et al., 2001; Takada et al., 2010).

*Haemophilus parainfluenzae* is a part of the normal flora of the human upper respiratory tract but has also been isolated occasionally from an increasing number of disease situations including meningitis, septicaemia, pleural effusion, urethritis, prosthetic joint infection, an abscess following reconstruction for facial paralysis, and endocarditis in patients with and without underlying heart disease (Bailey et al., 2011; Black et al., 1988; Cremades et al., 2011; Darras-Joly et al., 1997; Lee et al., 2012; Lin et al., 2012; Sturm, 1986). We recently showed that in contrast to the closely related species *Haemophilus influenzae*, *H. parainfluenzae* does not phase vary the expression of its core LPS components by the tetranucleotide repeat mediated slippage of LPS biosynthesis genes (Young and Hood, 2013) and at least one strain expresses polymeric OAg (Vitiazeva et al., 2011). The latter observation concurs with the findings of Roberts and colleagues (1986) that some (8/25) *H. parainfluenzae* isolates give ladder-like LPS profiles using silver-stained SDS-PAGE, suggestive of molecules containing OAgs of different chain lengths. As *H. parainfluenzae* OAgs have never been studied in detail, the number of serotypes is unknown and no antiserum is available to test for particular OAg structures. The aim of our research was to determine whether all *H. parainfluenzae* strains contains the genes necessary for OAg production, how the OAgs of different strains are related, and whether the OAgs play a role in bacterial–host interactions.

The mechanisms of OAg biosynthesis in other species have been well characterised. Whereas core LPS oligosaccharides are assembled onto lipid A-Kdo through the sequential transfer of each sugar from its nucleotide sugar precursor, the OAg polysaccharide is always added en bloc. An undecaprenyl phosphate (UndP)-sugar phosphotransferase transfers the first sugar of the OAg onto an UndP carrier lipid, and further glycosyltransferase enzymes add the subsequent sugars from their nucleotide sugar precursors. One of two alternative mechanisms is usually then used to polymerise and translocate the units (reviewed by Samuel and Reeves (2003)). In the Wzy-dependent system, the OAg flippase enzyme (Wzx) flips individual UndP-linked O-units from the cytoplasmic face to the periplasmic face of the inner membrane. The units are then polymerised by the OAg polymerase, Wzy, and the resulting OAg chain is ligated to the LPS core by the OAg ligase, WaaL. In this system, the modal chain length is determined by a fourth enzyme named Wzz. The alternative system requires an ABC transporter comprising two permease subunits (Wzm) for translocation and two ATPase subunits (Wzt) to drive the process. In this case the entire OAg chain is assembled on the cytoplasmic face of the inner membrane using glycosyltransferases before its translocation to the periplasmic side. The OAg is then ligated to the LPS core by WaaL as before. It is not known whether *H. parainfluenzae* uses one of these common mechanisms for OAg biosynthesis.

The enzymes required for OAg synthesis and assembly are usually encoded by a distinct, co-regulated gene cluster termed the OAg locus. The combination of OAg enzymes expressed by a particular bacterium determines the nature, order and linkages of the sugars in its O-unit, so analysis of LPS biosynthesis genes can greatly aid prediction of the OAg structure. The genetics of OAg biosynthesis in *H. parainfluenzae* have never been investigated.

In this paper we identify an OAg locus in the complete genome sequence of one of our *H. parainfluenzae* carriage isolates, strain T3T1. Investigation of the same region of the genome in 17 other diverse *H. parainfluenzae* carriage isolates using long range PCR and DNA sequencing reveals that the presence of an OAg gene cluster appears to be a ubiquitous feature of this species. Some OAg genes could also be amplified from two ‘hybrid’ strains included in our analyses; these two isolates have characteristics of both *H. parainfluenzae* and *H. influenzae* (Power et al., 2012; Young and Hood, 2013). Functional studies indicate a role for the OAg in the interaction between *H. parainfluenzae* and host cells or components of the immune system. This study of commensal *H. parainfluenzae* OAg loci and the corresponding OAg structures also lays the groundwork for future serotyping and genotyping classification schemes that would enable researchers to assess the distribution of disease isolates across the range of OAgs found in carriage strains.

## Materials and methods

### *Haemophilus* strains and culture

The *H. parainfluenzae* and *Haemophilus* hybrid strains were isolated from the throats of healthy children in the UK and The Gambia and have been numbered for convenience; full strain names are given in Table S7. Strains were grown in brain heart infusion broth (BHI) (Merck) supplemented with 2 μg/ml NAD and incubated at 37 °C for 16 h shaking at 200 rpm. For growth on solid medium, strains were plated on BHI agar (1%) supplemented with 10% Levinthals base (McLinn et al., 1970), which provides NAD, and incubated at 37 °C for 24 h.

### *Haemophilus* genomic DNA (gDNA) extraction

Bacteria from 3 ml log phase culture were pelleted by centrifuging at 13,000 ×* g* for 2 min then washed in PBS and resuspended in 200 μl TNE (100 mM NaCl, 10 mM Tris pH 8, 10 mM EDTA). SDS was added to 1%. Cells were lysed at 65 °C for 10 min then treated with proteinase K (500 μg/ml) at 37 °C for 2 h. The sample was then mixed with 1 vol phenol and centrifuged at 13,000 × *g* for 5 min; the top layer (containing DNA) was taken into a fresh tube and mixed with 1 vol phenol/chloroform/isoamyl alcohol (25:24:1). After centrifugation (13,000 × *g* for 5 min) the top layer was again taken into a fresh tube and the DNA was precipitated with 2 vol ethanol and 0.1 vol 3 M NaAc. The DNA was pelleted by centrifugation (13,000 × *g* for 10 min) and washed in 70% ethanol, then air dried and resuspended in 200 μl TE buffer (10 mM Tris pH 8, 1 mM EDTA) with 50 μg/ml RNase.

### Standard polymerase chain reaction (PCR)

For expected product sizes of up to 6 kb, 50 μl PCRs were prepared using 1 U Taq DNA Polymerase (Invitrogen). Each reaction also included 1× PCR Buffer (Invitrogen), approximately 40 ng template gDNA, 0.4 μM each primer (Sigma), 0.4 mM each dNTP and 2.5 mM MgCl_2_. DNA was amplified for 30 cycles comprising 1 min each of denaturation (94 °C), annealing (50 °C) and extension (72 °C); for expected products of >1.5 kb the extension time was increased to 3 min. PCR products were electrophoresed on 0.8% agarose gels containing 0.5 μg/ml ethidium bromide at 100 V for 1 h, and visualised under ultraviolet light. Primers used for PCR analysis are listed in Table S8.

### Long range PCR (LR-PCR) and digests

Fifty-microlitre LR-PCRs were performed using the Expand long range PCR kit (Roche) following the manufacturer's instructions. OAg loci were amplified from *H. parainfluenzae* gDNA using primers 5′-GAGACTGCGGTAGTCGATCC-3′ and 5′-CCATCACTTGGTTTGATGCT-3′, which are specific for the locus-flanking genes *glnA* and *pepB*, respectively. An extension time of 15 min in cycle 1, rising to 22 min by cycle 30, was found to be sufficient for the amplification of products of up to 20 kb. Five microlitres of each LR-PCR product was digested with MfeI (NEB). LR-PCR products and digests were run on 0.7% agarose gels at 20 V for 36 h.

### General cloning methods

Restriction enzymes (NEB) and T4 DNA ligase (Roche) were used as per the manufacturers’ instructions to construct recombinant plasmids. Plasmids were amplified by the transformation of chemically competent *E. coli* (Sambrook et al., 1989) and selection on LB agar (1% tryptone, 0.5% yeast extract, 0.5% NaCl, 1% agar) with the appropriate antibiotic (100 μg/ml ampicillin, 50 μg/ml kanamycin or 300 μg/ml erythromycin) and incubated at 37 °C for 24 h. Colonies were picked and plasmids extracted using the alkaline lysis method (Sambrook et al., 1989). The presence and orientation of the plasmid insert was determined by digestion with appropriate restriction enzymes.

### Disruption of *lgtF*, *waaL*, *wbaP* and *wcfS* genes

To disrupt specific LPS biosynthesis genes, the target region of DNA was amplified from *Haemophilus* gDNA by standard PCR and cloned in *E. coli* using the pSCA or pSCA-amp-kan vector system (Stratagene) following manufacturer's guidelines. The plasmid was cut with a restriction enzyme specific for a sequence in the reading frame to be disrupted and a drug resistance cassette with compatible ends was inserted. Uptake signal sequences (USS) were also included in some constructs with the aim of facilitating uptake of the plasmid DNA by *H. parainfluenzae*. The inclusion of USS in a plasmid appears to increase transformation rates in *H. influenzae* (Mitchell et al., 1991) and the same USS is also found throughout the *H. parainfluenzae* T3T1 genome. Plasmids are described in Table S10 and the primers used in their construction are listed in Table S11.

### Transformation of *H. parainfluenzae*

The success of various transformation methods was found to be highly strain-dependent, and the method used to generate each mutant is detailed in Table S9. Each transformation was first attempted with 0.5 μg linearised plasmid DNA using the static aerobic incubation method of Gromkova and Goodgal (1979), with 20 mM MgSO_4_. After shaking at 37 °C for 5 h, the transformation mixture was plated onto BHI with appropriate antibiotic selection (15 μg/ml kanamycin or 20 μg/ml erythromycin) and incubated at 37 °C for 24 h. Putative transformant colonies were checked for the mutant genotype using PCR analysis. If this did not yield transformants in the desired strain background, the same method was used but with the DNA source as 3 μg chromosomal DNA from a mutant from a different strain background that had been successfully transformed. To reduce the risk that extra recombination events between the donor and recipient genomes could affect phenotypic results, at least three independent clones were analysed whenever chromosomal donor DNA was used for transformation.

The genome sequence strain, *H. parainfluenzae* T3T1, could not be transformed using the static aerobic method but an electroporation protocol adapted from that of Mason et al. (2003) was successful. Fifty millilitres of BHI broth inoculated with an overnight culture to give a starting OD_600_ measurement of 0.10 was incubated at 37 °C with shaking. When the culture had reached an OD_600_ of 0.35 (150 min) it was chilled on ice for 30 min. All further steps were carried out at 4 °C. Cells were pelleted for 10 min at 4200 × *g* and washed 3 times with 0.5× SG (1× = 15% glycerol, 272 mM sucrose, pH 7.4) to increase their competence. After the final centrifugation, cells were resuspended in 500 μl 1× SG. Forty microlitres of competent cells were mixed with 1 μg circular plasmid DNA and were subjected to electroporation at 2.5 kV, 200 Ω and 25 μF (BioRad Gene Pulser), with recovery in 1 ml BHI for 90 min at 37 °C with shaking. Transformations were plated on BHI agar with antibiotics as described above.

### Cloning and sequencing OAg loci

OAg loci were amplified from *H. parainfluenzae* strains 13, 17, 20 and 30 using LR-PCR (see above). Each 12–19 kb product was digested using EcoRV and HaeIII in separate reactions then cleaned by ethanol precipitation and dissolved in H_2_O. The digested fragments were ligated to HincII-cut, phosphatase-treated pBluescript (Stratagene) with T4 DNA ligase (Roche) and cloned in *E. coli* DH5α. Colonies were selected on LB agar + 100 μg/ml ampicillin with 40 μg/ml X-Gal (5-bromo-4-chloro-3-indolyl-beta-d-galacto-pyranoside) to allow blue/white screening of inserts. Colonies were picked into 1 ml LB + 100 μg/ml ampicillin and grown for plasmid extraction. Restriction digestion using EcoRI and XhoI allowed clones to be categorised according to their insert size; one clone containing each EcoRV or HaeIII restriction fragment was then sequenced using primers M13-for-20 (5′-GTAAAACGACGGCCAGT-3′) and M13-rev-24 (5′-AACAGCTATGACCATG-3′) which bind to the pBluescript part of each construct and read into the insert region. The overlapping EcoRV and HaeIII fragment sequences were assembled into contigs using Vector NTI ContigExpress (Invitrogen), with additional PCR and sequencing across the gaps between contigs enabling full assembly of the OAg loci sequences. For completion of the strain 30 locus sequence, a second round of cloning was carried out using XmnI and SspI fragments, which were then sequenced as above. All DNA sequencing was carried out by the Weatherall Institute of Molecular Medicine Sequencing Service, John Radcliffe Hospital, Oxford, using an ABI-3730 DNA analyser with BigDye Terminator v3.1 (Applied Biosystems).

### Tricine SDS-PAGE for visualisation of LPS

*H. parainfluenzae* colonies were resuspended in PBS to equalised optical densities (approximately 10^9^ cells/ml). The suspensions were diluted 1:1 in 2× dissociation buffer (125 mM Tris pH 6.8, 20% glycerol, 4% SDS, 10% mercaptoethanol, 0.004% bromophenol blue). Following proteinase K treatment (50 μg/ml) at 60 °C for 3 h and denaturation at 100 °C for 5 min, 30 μl samples were fractionated on tricine SDS-PAGE gels (Lesse et al., 1990). LPS was visualised by staining with silver (GE Healthcare) following the manufacturer's instructions.

### Resistance to the bactericidal effects of human sera

The survival of *H. parainfluenzae* strains in human sera pooled from 15 to 18 donors was analysed using a method similar to that of Hood et al. (1999). Colonies grown on BHI agar were suspended in PBS-BG (PBS + 0.1% glucose (wt/vol) + 0.05 mM MgCl_2_ + 0.09 mM CaCl_2_), and a 1/20 dilution was prepared in 1% SDS, 0.1 M NaOH to measure the optical density at 260 nm. The starting suspension was adjusted to the equivalent of OD_260_ = 0.8 then diluted 1/20,000-fold. 20% pooled human serum (PHS) in PBS-BG was serially diluted 1:1 across seven wells of a flat bottomed polystyrene 96 well plate so that each well contained 50 μl of 20–0.32% PHS; the eighth well contained 50 μl of 20% PHS that had been decomplemented by heating at 56 °C for 30 min. Fifty microlitres of the diluted bacterial suspension (approximately 2000 c.f.u.) was added to each well, giving final PHS concentrations of 10–0.16%. After 1 h at 37 °C, 25 μl from each well was spread on BHI agar and incubated for 24 h at 37 °C. The resulting colonies were counted to determine the level of bacterial survival in each concentration of PHS.

### Epithelial cell association assay

The ability of *Haemophilus* strains to adhere to an SV40-transformed human bronchial epithelial cell line, 16HBE14o^−^, was studied using a protocol similar to that of Hood et al. (1999). This cell line has previously been used to study other respiratory tract bacteria. 16HBE14o^−^ cells were grown to a confluent monolayer in a flat bottomed polystyrene 96 well plate and washed three times with Hank's balanced salt solution (HBSS; Gibco). Fifty microlitres of 4% decomplemented PHS in Dulbecco's modified Eagle medium (DMEM; Gibco) was added to each well. Bacteria grown on BHI agar were resuspended in 1.5 ml PBS-BG and left to settle for 2 min before the top 1 ml was taken into a fresh tube; this avoids large clumps of cells. A suspension equivalent to OD_260_ = 0.8 was made using the method outlined in the section above, except that the cells were diluted with DMEM. Fifty microlitres of the bacterial suspension (approximately 4 × 10^7^ bacteria) was added to three monolayer wells and to three control wells containing no epithelial cells, and the plate was incubated for 2.5 h at 37 °C in the presence of 5% CO_2_. To measure association, all wells were washed with HBSS three times to remove non-adherent bacteria. 1% saponin was added to release the remaining bacteria (10 min at 37 °C), which were then serially diluted and plated on BHI agar. Colonies were counted after overnight incubation. Association was plotted as a percentage of the total number of bacteria present in a control monolayer well after the 2.5 h incubation period (i.e. adhered plus non-adhered bacteria).

To measure bacterial uptake by the epithelial cells, monolayers were incubated with the bacteria as above, then the medium was replaced with 250 μl of 200 μg/ml gentamicin. After a further 1.5 h at 37 °C the cells were washed, treated with saponin and plated as before. Gentamicin kills any bacteria exposed on the surface of the epithelial monolayer (this was confirmed by a sensitivity test) whilst internalised bacteria are protected from the antibiotic.

### Bioinformatic analysis

The Artemis genome browser and annotation tool (Rutherford et al., 2000) was used to examine *Haemophilus* genomes and OAg locus sequences. Sequence homology analysis was performed using the NCBI basic local alignment search tool (BLAST) (Altschul et al., 1997) with the default algorithm parameters. Conserved protein domains were detected using CDD (Marchler-Bauer et al., 2009). Transmembrane domains within proteins were predicted using the TMHMM V2.0 tool at www.cbs.dtu.dk/services/TMHMM.

### Sequence data

*H. parainfluenzae* sequence data have been submitted to the GenBank database under the following accession numbers: strain 13 OAg locus, KC759394; strain 20 OAg locus, KC759396; strain 17 OAg locus, KC759395; strain 30 OAg locus, KC759397; strain 19 *waaL* (external to the OAg locus), KC416614; strain 13 *waaL* (external to the OAg locus), KC416615; strain 19 *wzz* (within the OAg locus), KC416616.

## Results

### Many *H. parainfluenzae* strains exhibit OAg-like LPS patterns

**Fig. 1:**
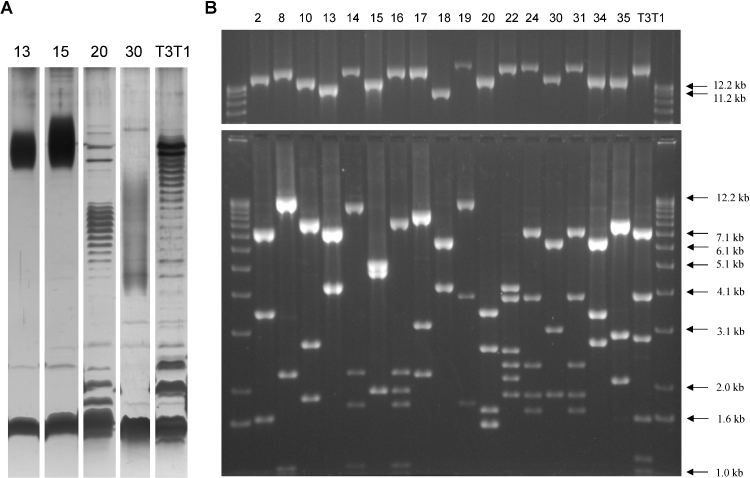
Visualisation of LPS and PCR investigation of potential OAg loci in *H. parainfluenzae*. (A) Proteinase K treated *H. parainfluenzae* cell lysates were fractionated by tricine SDS-PAGE and silver-stained. Strain numbers are listed above each lane. The intense low molecular mass band in each lane is LPS that does not contain OAg (LPS core only), whilst the ladders/smears of bands represent LPS elaborated with OAg of increasing chain length. Band spacing depends on the size of the O-unit. 12.5 μl of lysate at OD_260_ = 5 (strains 13 and 15) or OD_260_ = 10 (strains 20, 30 and T3T1) was loaded. Weaker OAg-like banding patterns were observed for strains 2, 8, 10, 14, 16, 17, 18 and Hy6. (B) Long range PCR products were obtained using primers to *glnA* and *pepB*, which flank the OAg locus. The outside lanes contain a DNA ladder with sizes as indicated. Numbers above each lane indicate the *H. parainfluenzae* strain of the template gDNA. Top panel: 0.4 μl of each PCR reaction separated by agarose gel electrophoresis. Products of a high molecular mass are visible for all 18 true *H. parainfluenzae* strains; hybrid strains Hy6 and Hy11 did not yield products and are not shown. Lower panel: MfeI restriction digests of the same long range PCR products, with fragments >1 kb visible.

The presence of OAg was investigated in the LPS of 18 *H. parainfluenzae* carriage isolates from healthy children in the UK and The Gambia and two ‘hybrid’ strains (Hy6 and Hy11) using silver-stained tricine SDS-PAGE analysis of proteinase K treated cell lysates. Evenly-spaced ladders of bands typical of a repeating oligosaccharide unit (OAg) were detected in the LPS profiles of 13/20 (65%) of the isolates. It is likely that these bands correspond to LPS core plus OAg of varied chain length. The intensity, apparent O-unit size and average molecular weight of the putative OAg glycoforms are reproducible for each strain under laboratory growth conditions but vary greatly between strains (Fig. 1A and Fig. S1), suggesting differences in O-unit composition and chain length regulation.

### All *H. parainfluenzae* strains contain a putative OAg locus between *glnA* and *pepB*

Analysis of the genome sequence of one of our OAg-expressing strains, T3T1, the first *H. parainfluenzae* strain to have its genome sequenced and fully assembled (Wellcome Trust Sanger Institute, Cambridge) (EMBL accession number FQ312002), reveals a putative OAg biosynthesis locus comprising 16 genes (PARA_02720–PARA_02870) over 16.77 kb (99.2% coding). This gene cluster is flanked by the *glnA* and *pepB* genes. In many species of Gram negative bacteria the OAg locus is at the same genomic location in different strains, and indeed the *hmg* (high molecular weight glycoform) locus that is responsible for the addition of a single tetrasaccharide unit to the LPS in some *H. influenzae* isolates is also located between *glnA* and *pepB* (Hood et al., 2004). To investigate whether other *H. parainfluenzae* strains carry an OAg locus at this location, long range PCR (LR-PCR) was carried out using gDNA from each of the 20 study strains with primers designed to the *H. parainfluenzae* T3T1 *glnA* and *pepB* genes.

PCR products ranging from 12 to 19 kb in length were obtained for 18/18 true *H. parainfluenzae* strains (Fig. 1B, upper panel), suggesting that a series of genes consistently falls between *glnA* and *pepB* in this species. No products were obtained for the two hybrid strains (Hy6 and Hy11). As the ends of the PCR products contain part of the flanking genes, the actual size of the putative OAg loci was predicted to be 10–17 kb, with the T3T1 locus amongst the largest. MfeI restriction digest profiles of the 18 LR-PCR products were almost all unique, indicating a high level of nucleotide divergence between the loci (Fig. 1B, lower panel). Only *H. parainfluenzae* strains 24 and 31 had identical restriction profiles.

### Sequencing of the putative OAg locus of four isolates

**Fig. 2:**
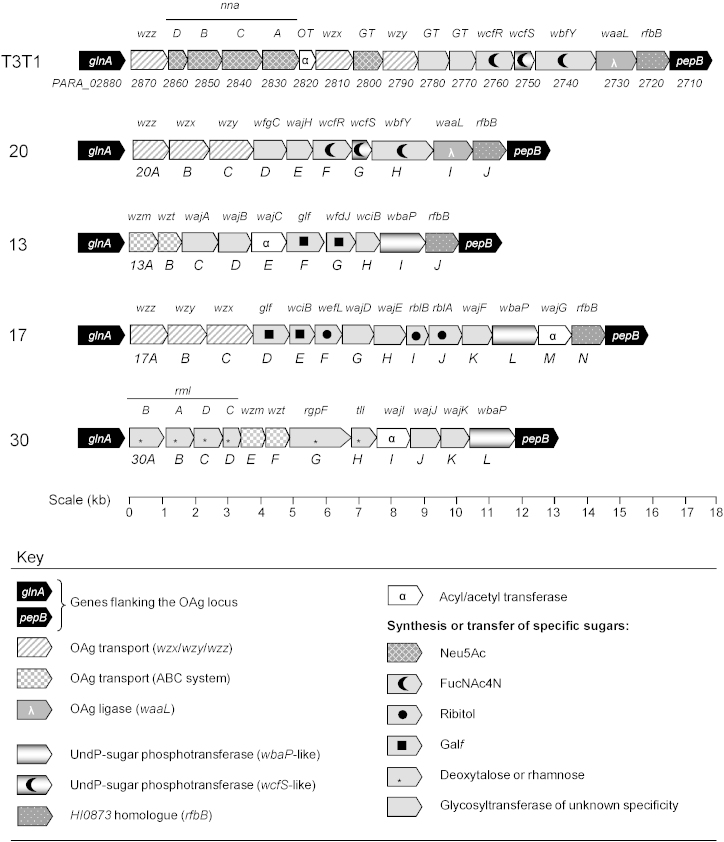
Organisation of the five sequenced *H. parainfluenzae* OAg loci. The strain name is given to the left of each diagram. Each block arrow represents an ORF, and its predicted function (as discussed in the text) is indicated by its shading and pattern as shown in the key. Each locus encodes one of two transport systems (Wzy-dependent or an ABC transporter), an UndP-sugar phosphotransferase, and a series of enzymes for the synthesis and transfer of various OAg components. Drawn to the scale indicated. GT = glycosyltransferase, OT = O-acetyltransferase.

The *glnA*–*pepB* PCR product from four of the *H. parainfluenzae* isolates (strains 13, 17, 20 and 30) was digested, cloned using *E. coli* plasmid vectors and sequenced to investigate whether they encoded putative OAg biosynthesis enzymes. The reasons for selecting these strains are detailed later. Each assembled sequence comprised 10–14 open reading frames in the *glnA* to *pepB* orientation. There is very little intergenic DNA, suggesting that in general the genes at each locus form an operon in which the genes are co-regulated and co-transcribed. The putative role of each encoded protein in these four loci and in the *H. parainfluenzae* T3T1 OAg locus was explored by comparison to homologues of known function, conserved domain searches and in some cases simple tertiary structure modelling. Using these methods it was possible to predict parts of each OAg structure through bioinformatics alone. The five loci all encoded enzymes with predicted functions in nucleotide sugar biosynthesis, sugar transfer and OAg assembly and transport, but detailed analysis predicted highly diverse sugar structures and methods of assembly as described below (Fig. 2). New gene names (*wajA*–*wajK*) were obtained from the curators of the Bacterial Polysaccharide Gene Database (http://sydney.edu.au/science/molecular_bioscience/BPGD/) for 11 of the predicted glycosyltransferase and acyltransferase genes.

### *H. parainfluenzae* T3T1 synthesises a tetrasaccharide O-unit using the Wzy-dependent system

The proteins encoded by genes *PARA_02760*, *PARA_02750* and *PARA_02740* (Fig. 2 and Table S1) are similar to the three enzymes in the proposed pathway for the biosynthesis of UndP-linked FucNAc4N in *E. coli* Sonnei, using UDP-GlcNAc as the precursor (Xu et al., 2002). This suggested that the first sugar of the *H. parainfluenzae* T3T1 O-unit could be FucNAc4N (also known as 2-acetamido-4-amino-2,4,6-trideoxygalactose or AAT), a hypothesis that was confirmed by subsequent structural analysis of LPS containing a single O-unit (data not shown; Twelkmeyer et al., manuscript in preparation). Similar genes are also found in *Bacteroides fragilis*, where FucNAc4N is the first sugar of the repeat unit for the polysaccharide A (PS-A) capsule (Baumann et al., 1992; Coyne et al., 2001). FucNAc4N is a rare sugar that has been identified as part of various structures in only a few other bacterial species to date, namely the *Streptococcus pneumoniae* serotype 1 capsule (Bentley et al., 2006), *S. pneumoniae* and *Streptococcus mitis* lipoteichoic acid (Bergstrom et al., 2000; Draing et al., 2006), and OAg or OAg-core linker structures in *E. coli* Sonnei, *Plesiomonas shigelloides*, *Bordetella* species and *Proteus vulgaris* (Arbatsky et al., 2007; Kenne et al., 1980; Preston et al., 2006; Shepherd et al., 2000). BLASTP searches of sequences available for these species (shaded in Table S1) suggest that the FucNAc4N biosynthesis pathway is highly conserved, and one might predict that other bacteria with these genes such as *Porphyromonas endodontalis* and *Fusobacterium nucleatum* may also synthesise FucNAc4N as part of a glycoconjugate.

The proposed UndP-sugar phosphotransferase PARA_02750 shares some sequence similarity (33–37% aa identity) with the C-terminal end of the UndP-Gal phosphotransferase (WbaP) enzymes from *Salmonella* and *H. influenzae*, but as it appears to add FucNAc4N rather than Gal as the initial sugar of the O-unit we will refer to it as WcfS, after the UndP-FucNAc4N phosphotransferase from *B. fragilis* (72% aa identity). In addition to PARA_02750, which contains a predicted transmembrane domain, the strain T3T1 OAg locus encodes two putative cytoplasmic glycosyltransferases: PARA_02770 and PARA_02780. These are likely to add the second and third sugars of the O-unit, known to be Gal and GalNAc respectively, but the sugar specificity of each enzyme is unclear.

Several proteins encoded by the *H. parainfluenzae* T3T1 OAg locus are predicted to relate to the metabolism and transfer of sialic acid (Neu5Ac), a common component of OAg and capsular polysaccharides (Fig. 2 and Table S1). The substrate for sialic acid addition is usually the nucleotide sugar CMP-Neu5Ac, whose biosynthetic pathway from UDP-GlcNAc requiring the four enzymes NnaA–NnaD has been well characterised for *E. coli* (Vimr et al., 2004). Putative *nnaA*–*nnaD* genes are present within the T3T1 OAg locus (*PARA_02830*–*PARA_02860*), sharing high levels of sequence similarity with the genes for polysialic acid capsule biosynthesis in *Mannheimia haemolytica* serotype A2 strains (Adlam et al., 1987). In addition, PARA_02800 is a putative Family 52 glycosyltransferase that shares 40% aa identity with well-characterised capsule sialyltransferases from *M. haemolytica* serotype A2 and *Streptococcus agalactiae* serotype VIII strains. In agreement with these observations, the fourth sugar of the *H. parainfluenzae* T3T1 O-unit was found to be acetylated Neu5Ac (Twelkmeyer et al., manuscript in preparation). The acetylation of the sialic acid residue might be carried out by either NnaD (NeuD; PARA_02860) or the putative O-acetyltransferase PARA_02820.

*H. influenzae* can also decorate its LPS with Neu5Ac, which it obtains from the environment using a tripartite ATP-independent periplasmic (TRAP) transporter encoded by the *siaP* and *siaQ/M* genes (Severi et al., 2005). The ability of *H. parainfluenzae* strain T3T1 to synthesise Neu5Ac obviates the need to import this sugar, and indeed there are no *siaP* or *siaQ/M* homologues in the T3T1 genome.

The Wzy-dependent pathway of OAg assembly and transport requires an OAg flippase (Wzx), OAg polymerase (Wzy), chain length determinant (Wzz) and OAg ligase (WaaL). The *H. parainfluenzae* T3T1 OAg locus appears to encode enzymes with each of these functions (Fig. 2 and Table S1). A conserved domain search predicts PARA_02810 to belong to the RfbX family of membrane proteins involved in OAg export, whilst the TMHMM transmembrane modelling algorithm predicts that it contains 12 transmembrane domains, typical of OAg flippase enzymes. The highest scoring BLASTP matches for PARA_02790 are OAg and capsular polysaccharide polymerases from a range of bacterial families. OAg polymerases typically show little amino acid similarity to each other but their tertiary structure is more conserved, with at least 10 transmembrane domains anchoring the protein in the inner membrane (Kim et al., 2010). TMHMM predicts PARA_02790 to contain 10 transmembrane helices.

The chain length distribution of *H. parainfluenzae* T3T1 OAg is bimodal, with most LPS molecules having either 0–2 or around 20 O-units (Fig. 1A). Its OAg chain length determinant Wzz, encoded by gene *PARA_02870*, is most closely related to those of other Pasteurellaceae genera that produce OAg including several *Mannheimia* and *Actinobacillus* species. The topology predicted for PARA_02870 by TMHMM is a long periplasmic loop flanked by two transmembrane domains, fitting the structure that has been determined for several polysaccharide co-polymerases including Wzz of other species (Morona et al., 2009).

The *H. parainfluenzae* T3T1 OAg locus has some notable characteristics when compared to the genome as a whole. Whilst the average G + C content of the genome is 39.6%, that of the OAg locus is only 32.1%; this low %G + C is typical of OAg loci in Gram negative bacteria. In addition, the 9 bp *H. influenzae* uptake signal sequence (USS) (Redfield et al., 2006) is distributed throughout the T3T1 genome at an average frequency of one every 1.4 kb but is absent from the OAg locus. These observations support the hypothesis that *H. parainfluenzae* has acquired this region of DNA through horizontal gene transfer events relatively recently.

### The *H. parainfluenzae* strain 20 OAg contains a phosphate linkage whose formation is catalysed by a ‘Stealth’ protein

Intragenic PCR amplification was performed on gDNA from the 20 study strains to test for the presence of some of the *H. parainfluenzae* T3T1 OAg locus genes (Table 1). PCR using primers designed to the putative UDP-GlcNAc dehydratase gene, *PARA_02740*, amplified products for 7/20 strains. This indicated that several strains may be capable of decorating their LPS with at least one of the same sugars as strain T3T1, namely FucNAc4N. In parallel with the determination of the OAg structure from one of these strains, *H. parainfluenzae* 20 (Vitiazeva et al., 2011), we sequenced and analysed its full OAg locus. This strain was chosen for analysis due to its high level of OAg expression and because its short OAg locus indicated a different genetic composition to that of T3T1.

Upon assembly, the DNA sequence was found to comprise 10 intact genes spanning 11.7 kb (Fig. 2). The products of seven of these ORFs share 68–99% aa identity with proteins encoded by the strain T3T1 OAg locus (Table S2). The predicted chain length determinant protein, 20A, shares 83% aa identity with that of *H. parainfluenzae* T3T1, reflecting the similar modal OAg chain length of the two strains (Fig. 1A). However, the predicted Wzx (20B) and Wzy (20C) proteins share so little identity with the strain T3T1 alleles that they cannot be aligned; like the FucNAc4N biosynthesis genes they are closely related to enzymes from *Bacteroides* and may have been acquired together through horizontal gene transfer.

In addition to the WcfS protein, which determines the first sugar of the O-unit as FucNAc4N, the *H. parainfluenzae* strain 20 OAg locus encodes two other transferases (20D and 20E). Protein 20D shares 37% aa identity with WfgC encoded by the OAg loci of *E. coli* serogroup O152 and *Shigella dysenteriae* group 12. These two OAg contain the moiety α-Glc*p*NAc-(1 → *P *→ 6)-α-Glc*p* (Liu et al., 2008; Olsson et al., 2005), and WfgC is predicted to catalyse the unusual phosphodiester linkage between the two sugars (Lundborg et al., 2010). In 2005, Sperisen and colleagues identified a novel family of proteins that was conserved across most eukaryotes and some prokaryotes (Sperisen et al., 2005). They termed it ‘Stealth’ because the bacterial members of the family appeared to be involved in immune evasion, and hypothesised that the proteins were hexose-1-phosphoryl transferases. When compared to the Stealth alignment published by Sperisen et al., it becomes clear that protein 20D belongs to this family. The conserved regions (CR) which define Stealth are present at the following positions within the 20D sequence: CR1 = aa 5–16; CR2 = aa 40–139; CR3 = aa 223–271; CR4 = aa 308–343. Together with its similarity to WfgC, this information strengthens the case for gene *20D* encoding a phosphoryl transferase (i.e. an enzyme that connects two sugars via a phosphodiester linkage, as seen in the strain 20 O-unit structure). The presence of the three transferase genes and the lack of sialic acid biosynthesis/transfer genes in the strain 20 OAg locus is consistent with the observed trisaccharide O-unit structure, which contains FucNAc4N, glucose phosphate and GalNAc (Vitiazeva et al., 2011).

We have recently shown that deleting *wcfS* in *H. parainfluenzae* strain 20 results in both the loss of a ladder pattern on the SDS-PAGE LPS profile and the loss of detectable OAg using structural analysis, confirming the involvement of this gene in OAg production (Vitiazeva et al., 2011).

### *H. parainfluenzae* strain 13 uses an ABC transporter to add heteropolymeric OAg to LPS

*H. parainfluenzae* strain 13 was chosen as the next isolate for analysis because its OAg has a different chain length distribution and O-unit size to that of strains T3T1 or 20 (Fig. 1A), its *glnA*–*pepB* LR-PCR product is intriguingly short (Fig. 1B), and it was negative by PCR amplification for gene *PARA_02740* suggesting an OAg structure that may not contain FucNAc4N. Following cloning and sequencing of the *glnA*–*pepB* region, BLASTP searches of the 10 ORFs found predicted an almost entirely different set of enzymes to those encoded by the *H. parainfluenzae* T3T1 locus, with only two genes bearing any similarity between the strains. However, the strain 13 ORFs still encode typical OAg synthesis proteins including nucleotide-sugar synthesis enzymes, glycosyltransferases and an OAg transport system (Fig. 2 and Table S3).

Gene *13F* encodes a protein with significant homology to the Glf family of UDP-galactopyranose mutases (Pfam03275). Glf catalyses the conversion of UDP-galactopyranose to UDP-galactofuranose so that galactofuranose (Gal*f*) can be incorporated into structures such as the mycobacterial cell wall (Pan et al., 2001) or *E. coli* OAg (Nassau et al., 1996). The different conformations of Gal*p* (6-membered ring) and Gal*f* (5-membered ring) may confer different biological properties on the resulting structures; Gal*f* is thermodynamically less stable and occurs much less frequently in nature. The presence of *glf* within the *H. parainfluenzae* strain 13 OAg locus strongly suggested the presence of Gal*f* or a derivative in the O-unit, and this was confirmed by structural analysis (data not shown; Twelkmeyer et al., manuscript in preparation). The Gal*f*-(1,3)-β-d-Glc*p*NAc linkage found in the disaccharide O-unit is likely to be formed by the glycosyltransferase encoded by gene *13G*, as this is the exact predicted sugar and linkage specificity of *E. coli* WfdJ with which it shares 36% aa identity (Lundborg et al., 2010). The specificities of the three other predicted glycosyltransferases (WajA, WajB and WciB) and of the WbaP-like protein 13I have not been determined.

The strain 13 OAg structure also contains PEtn and O-acetyl (OAc) substituents. LPS O-acetylation helps to confer resistance to antimicrobial peptides in some species (Gunn, 2001). OAc is likely to be added to Gal*f* by the predicted O-acetyltransferase WajC (Pfam01757), but no PEtn transferase was identified in the OAg locus. The HMG unit in *H. influenzae* is decorated with PEtn by an enzyme encoded outside the *hmg* locus (Derek Hood, unpublished observations) and a similar situation may occur in *H. parainfluenzae* strain 13. In species such as *Bordetella bronchiseptica* and *Shigella flexneri*, certain OAgs are known to undergo late modification by OAc or PEtn transferases after the chain has been transported to the periplasm (Allison and Verma, 2000; King et al., 2009).

Strain 13 evidently uses an ABC2 transporter, rather than Wzx, to transfer completed OAg from the cytoplasmic to the periplasmic face of the inner membrane. The permease and ATPase subunits (Wzm and Wzt) are encoded by the first two ORFs of the OAg locus and are closely related to enzymes encoded by polysaccharide biosynthesis gene clusters in *Actinobacillus pleuropneumoniae* and *Aggregatibacter aphrophilus*, also members of the Pasteurellaceae family. Modal chain length regulation in ABC transporter dependent OAg systems is poorly understood; in some *E. coli* serotypes, chain termination and transport occurs upon methylation of the terminal sugar (Clarke et al., 2004). No methylated sugars were found in the *H. parainfluenzae* 13 OAg by structural analysis (Twelkmeyer et al., manuscript in preparation) and none of the proteins encoded by its OAg locus is predicted to contain the coiled coil motif that is typical of methyltransferases, suggesting that the tight OAg chain length distribution in this strain is controlled by a different mechanism. Following the ABC transporter nucleotide-binding domain classification scheme of Cuthbertson et al. (2010), the *H. parainfluenzae* strain 13 Wzt protein falls into phylogenetic group D. Members of this group lack C-terminal extensions and the polysaccharides that they transport do not typically contain chain-terminating modifications.

### Most *H. parainfluenzae* strains have a T3T1-like (group 1) or a strain 13-like (group 2) OAg locus

To investigate whether other *H. parainfluenzae* strains have similar OAg loci to the three sequences that were now available, internal primers were designed to each gene in the *H. parainfluenzae* strain 13 OAg locus and to some of the genes that had yet to be studied from the *H. parainfluenzae* T3T1 OAg locus. The presence of each gene was tested in the study strains by PCR amplification (Table 1). The results were striking in that they immediately separated 15 of the 18 true *H. parainfluenzae* strains into two clear OAg locus categories. Seven strains, designated as group 1, contained homologues of the genes needed for FucNAc4N synthesis and transfer in strain T3T1 (genes *PARA_02760*, *PARA_02750* and *PARA_02740*). A subset of these seven strains also gave PCR products for the two putative strain T3T1 glycosyltransferase genes and/or the sialic acid synthase gene *nnaB* (*neuB*). The *nnaB*-positive strains correspond to the five largest LR-PCR products in Fig. 1B. Most strains in group 1 also showed evidence for genes required for the Wzy-dependent mechanism of OAg synthesis and transport (Table 1).

A mutually exclusive set of eight strains were positive for the *H. parainfluenzae* strain 13 UndP-sugar phosphotransferase gene *13I*, and for *13H* which we predict to encode an enzyme involved in the synthesis or transfer of Gal*f*. All of these isolates except strain 35 also appear to encode a similar ABC transporter (permease subunit) to that of the strain 13 OAg locus. The eight isolates containing the strain 13-like loci were designated as group 2. In addition the two hybrid strains, Hy6 and Hy11, share a few of the group 2 OAg locus genes. Group 1 and group 2 OAg loci are found in both Gambian and UK strains, but the group 2 loci from Gambian strains (*H. parainfluenzae* 13, 15 and 18) appear to be particularly closely related.

OAg locus mapping by PCR amplification of various combinations of adjacent genes indicated that the order of the genes that are present is generally conserved within the groups (RY, unpublished data). It is evident from Fig. 1B that the OAg loci of *H. parainfluenzae* strains 14 and 16 are several kilobases longer than that of strain 13; perhaps the central region comprises a novel set of glycosyltransferase genes and/or sugar synthesis genes instead of ORFs *13C* to *13E*.

No strains yielded PCR products for both group 1 and group 2 genes: although strains T3T1 and 13 share the last ORF in the OAg locus (*PARA_02720*/*13J*) there are no other genes in common, so the exchange of sections of the OAg locus between group 1 and group 2 strains by homologous recombination would be unlikely. The different assembly systems used by the two groups (Wzy-dependent or ABC transporter) would also impose constraints on reassortment, if the OAg locus is to remain functional.

For three strains (*H. parainfluenzae* 17, 30 and 34), no PCR products were obtained using any of the group-specific T3T1 or strain 13 primers. To investigate whether these strains contained novel sets of OAg genes, the *glnA*–*pepB* regions of *H. parainfluenzae* strains 17 and 30 were digested, cloned in *E. coli* and sequenced. Both of these strains produce unique OAg-like ladders visible on a heavily loaded SDS-PAGE gel (Fig. 1A and Fig. S1), suggesting that their OAg loci are likely to be both novel and functional.

### Analysis of the *H. parainfluenzae* strain 17 OAg locus

Following sequence assembly, the *H. parainfluenzae* strain 17 *glnA*–*pepB* region was found to contain 14 ORFs over 14.8 kb (Fig. 2). The genes encode functions that are typical of OAg synthesis and Wzy-dependent assembly (Table S4). In brief, the locus includes genes encoding six putative glycosyltransferases and one acyltransferase, suggesting an O-unit comprising up to six sugars and at least one OAc group. Two genes provide evidence for the presence of Gal*f* in the OAg. Firstly, the product of gene *17D* shares 87% aa identity with Glf from *S. pneumoniae*, where it is present in strains that include Gal*f* in their capsule. Secondly, gene *17E* is a putative homologue of pneumococcal *wciB*, whose product has been categorised as a Gal*f* transferase and always adds the sugar via a β1,3 linkage (Aanensen et al., 2007).

17I and 17J are similar to the enzymes required for the two-step conversion of d-ribulose-5P to CDP-ribitol (Baur et al., 2009), which is used to make the polyribitol-phosphate component of teichoic acid in the Gram positive cell wall. Protein 17F shares 61% aa identity with WefL in *S. oralis* strains C104 and SK144, where it is proposed to transfer ribitol-5-phosphate to Gal*f* (Yang et al., 2009). Ribitol phosphate is an unusual OAg component and would contribute negative charge to the O-unit.

### Analysis of the *H. parainfluenzae* strain 30 OAg locus

The *H. parainfluenzae* strain 30 *glnA*–*pepB* region contains 12 ORFs (Fig. 2). The first 11 of these correspond to the majority of the 12-gene *Aggregatibacter* (previously *Actinobacillus*) *actinomycetemcomitans* serotype c OAg locus, with 71–94% aa identity between gene products (Table S5). This greatly aids prediction of the *H. parainfluenzae* strain 30 OAg structure, as the *A. actinomycetemcomitans* serotype c OAg has been studied in detail and is known to comprise → 3)-6-deoxy-α-l-talose-(1,2)-6-deoxy-α-l-talose-(1→ with acetylation at the O-4 position of the first of these two sugars (Nakano et al., 1998; Shibuya et al., 1991).

Overall, the O-unit is likely to be very similar to that of *A. actinomycetemcomitans* serotype c, including one or more 6-deoxy-l-talopyranose residues and some degree of acetylation carried out by the product of gene *30I*. The OAg may also contain l-rhamnose, which differs from 6-deoxy-l-talose only in the stereochemistry of the C4 carbon. 6-deoxy-l-talose has previously been found in the OAg of three *E. coli* serotypes (Jann et al., 1995) and the *Mesorhizobium loti* type strain (Russa et al., 1995).

Primers designed to the strain 30 genes *30B* (rhamnose/deoxytalose synthesis pathway gene) and *30E* (ABC transporter permease subunit gene) amplified PCR products from *H. parainfluenzae* strain 34 gDNA (Table 1), and the MfeI digestion pattern of the LR-PCR product from this strain includes a fragment of approximately the same molecular mass as the *30G*–*30L* fragment from *H. parainfluenzae* 30 (6.5 kb; Fig. 1B). It therefore seems likely that strain 34 contains an OAg locus of a broadly similar composition to that of strain 30, although no OAg has been observed for the former strain. These results mean that putative OAg biosynthesis genes have now been detected in all 20 study strains.

### Recommended primers for the categorisation of *H. parainfluenzae* OAg loci

The diversity of OAg genes within the 20 strains of the study panel is so great that it is not practical to develop a serotype naming system at present. However, it may be useful for researchers to categorise clinical isolates broadly by OAg group and/or to compare the restriction patterns of their OAg loci to examine virulence trends. Group 1 and group 2 OAg loci may be distinguished by two simple polymerase chain reactions using gDNA; we recommend primer pair P20/P21, which amplifies a 656 bp fragment of a FucNAc4N biosynthesis gene in group 1 loci, and P37/P38, which amplify a 537 bp fragment of a Gal*f* biosynthesis gene in group 2 loci. Primer sequences are given in Table S8.

### Identification of an OAg ligase gene between *fba* and *orfH*, outside the OAg gene cluster

The transfer of OAg from the UndP carrier to core LPS is usually carried out by an OAg ligase. Whilst all seven group 1 *H. parainfluenzae* OAg loci include a putative OAg ligase gene (Table 1), the group 2 OAg locus of strain 13 and the ungrouped OAg loci of strains 17 and 30 do not. We therefore investigated whether any of the unsequenced *H. parainfluenzae* OAg loci contain an OAg ligase gene in a particular position and whether strain 13 or other strains encode an OAg ligase elsewhere in the genome.

In the *H. influenzae hmg* locus, the ORF encoding the HMG ligase is at the distal end between *HI0873* (annotated as *rfbB* but of unknown function) and the locus-flanking gene *pepB* and is convergent in orientation with the rest of the locus (Hood et al., 2004). PCR analysis demonstrated that a ligase gene is not found between *rfbB* and *pepB* in any of our 18 *H. parainfluenzae* strains (Fig. S2).

The *H. parainfluenzae* strain 13 genome has not been sequenced, but limited sequence data is available for strain 15 (Power et al., 2012), which produces OAg with a similar PAGE profile and has a similar OAg locus to strain 13 (Fig. 1A and Table 1). Using BLASTP we identified a DNA sequence fragment from strain 15 with similarity to the 3′ end of the *H. parainfluenzae* T3T1 OAg ligase gene *PARA_02730*. PCR amplification and further sequence analysis revealed that the ORF was flanked by the LPS HepI transferase gene *orfH* and the fructose bis-phosphate aldolase gene *fba* in strain 15; these two genes are adjacent in strain T3T1 (*PARA_08430* and *PARA_08440*, respectively).

PCR analysis was performed to test which *H. parainfluenzae* study strains contain a strain 15-like *waaL* gene. Using internal strain 15 *waaL* primers, a 730 bp product was amplified from 13 of the 20 study strains (Fig. S3). These comprised all eight *H. parainfluenzae* strains with group 2 OAg loci, the three ungrouped strains (17, 30 and 34), and two strains with group 1 OAg loci (2 and 19). PCR products obtained using primers designed to *fba* and *orfH* were consistent with the presence of an intervening gene (i.e. *waaL*) at this locus in the same 13 strains (Fig. S3).

Full length strain 15-like *waaL* DNA sequences were obtained for strains 13 and 19 and are available via Genbank. When translated, these sequences share more homology with WaaL from *A. aphrophilus* (57% aa identity) than with the *H. influenzae* Rd HMG ligase, HI0874 (42%) or the *H. parainfluenzae* T3T1 OAg ligase, PARA_02730 (34%). TMHMM analysis predicts that the strain 13 WaaL contains 12 membrane-spanning domains and an 89 aa periplasmic loop. This topology is typical for an OAg ligase: the periplasmic loop regions of *Salmonella enterica* sv. Typhimurium and *E. coli* WaaL are predicted to be 73 and 84 aa, respectively (Abeyrathne and Lam, 2007).

**Fig. 3:**
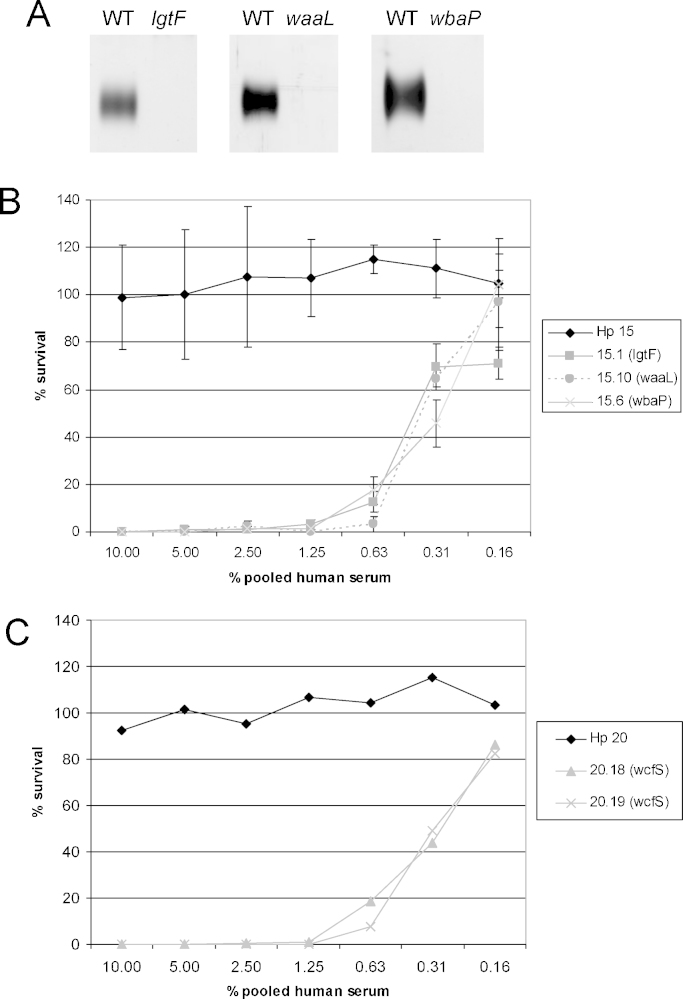
Phenotypic analysis of *H. parainfluenzae* OAg mutants derived from strains with group 1 or group 2 OAg loci. (A) LPS profiles of *H. parainfluenzae* strain 15 and its *lgtF*, *waaL* and *wbaP* mutants, showing the loss of OAg in all three mutants. 12.5 μl of proteinase K treated cell lysates at OD_260_ = 1 were separated by tricine SDS-PAGE and silver-stained. Only the OAg region of the gel is shown. (B) Resistance of *H. parainfluenzae* strain 15 (group 2) and its OAg mutants to the killing effect of pooled human serum. Results are shown as the survival of inoculating bacteria as a percentage of the survival in a 10% decomplemented serum control well. Each data point represents the mean of three replicates; error bars show ±standard error of the mean. (C) Resistance of *H. parainfluenzae* strain 20 (group 1) and its UndP-sugar phosphotransferase mutants to the killing effect of pooled human serum. Results are shown as the survival of inoculating bacteria as a percentage of the survival in a 10% decomplemented serum control well. 20.18 and 20.19 are independent transformants made using the same parental strain and plasmid. Each data point represents the mean of two replicates.

An *H. parainfluenzae* strain 15 mutant in which the newly discovered OAg ligase gene was disrupted did not synthesise any OAg-containing glycoforms (Fig. 3A), consistent with the hypothesis that this gene encodes the ligase responsible for the addition of the OAg to the LPS core in this strain.

Whilst it is reassuring to identify the OAg ligase gene in the group 2 and ungrouped *H. parainfluenzae* strains, its presence in strains 2 and 19 was unexpected as these also include genes related to *H. parainfluenzae* strain 20 *waaL* in their OAg loci (PCR analysis, Table 1). A possible scenario is that these two lineages recently exchanged a group 2 locus for a new group 1 locus, acquiring an extra OAg ligase gene in the process, and have not yet lost the original ligase gene. Alternatively, one might be a pseudogene, or the two ligases might act upon different donor (polysaccharide) and/or acceptor (LPS/protein) molecules in these strains. OAg ligase homologues are known to add O-linked sugars to certain cell surface proteins in some other species, e.g. pilin glycosylation in *Neisseria meningitidis* (Power et al., 2006).

### The O-antigen confers resistance to complement-mediated killing

Having established that all of the *H. parainfluenzae* strains tested contain an OAg gene cluster, we investigated the biological roles of the O-antigens using several in vitro assays that are proxies for aspects of host interactions. Serum isolated from human blood contains complement components and some antibodies, so the serum bactericidal assay can detect both classical and alternative complement activation. It primarily measures killing that is mediated by MAC formation, because serum does not contain the macrophages and neutrophils that are required for opsonophagocytosis.

The UndP sugar transferase genes in *H. parainfluenzae* strains 20 and 15 (*wcfS* and *wbaP*, respectively) were disrupted with a kanamycin resistance cassette by transformation with plasmid constructs. This resulted in the complete loss of OAg from both strain 20 (Vitiazeva et al., 2011) and strain 15 (Fig. 3A), demonstrating that the respective loci are required for OAg formation as predicted. Elimination of the OAg by disrupting *wcfS*, *wbaP*, *waaL* or *lgtF* results in a dramatic loss of resistance to the killing effect of complement in pooled human serum across a range of *H. parainfluenzae* strains, regardless of whether the OAg locus was categorised as group 1 or group 2 (Fig. 3B and C and Table S6).

### Epithelial adhesion is affected by extensions from HepI of the LPS

**Fig. 4:**
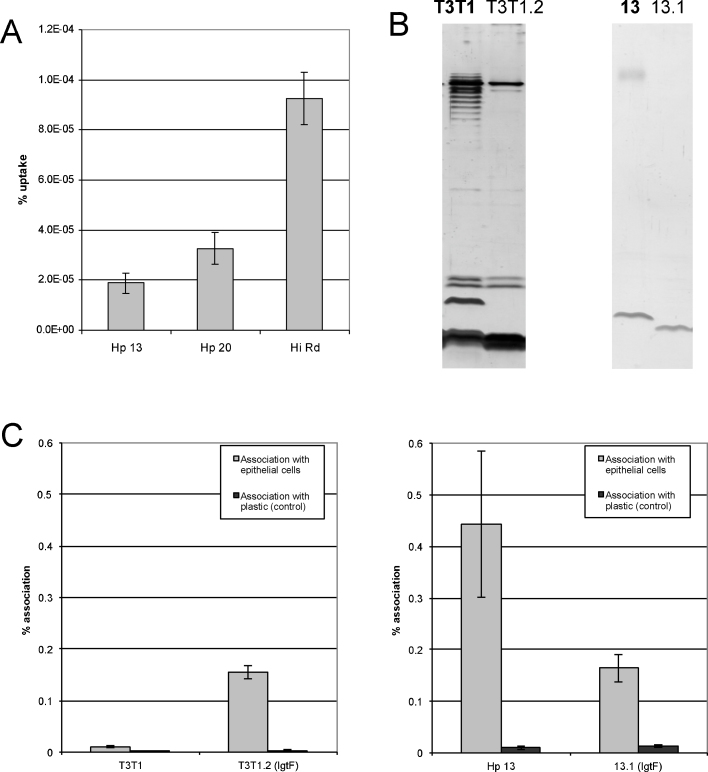
Interactions between epithelial cells and *Haemophilus* bacteria in vitro. (A) Uptake of *Haemophilus* bacteria by human bronchial epithelial cells. *H. parainfluenzae* strains 13 and 20 and *H. influenzae* strain Rd were incubated in a 96-well plate with a monolayer of 16HBE14 cells. The *y* axis records the number of bacteria that were inside the epithelial cells after a 2.5 h incubation as a percentage of the total bacteria in a control well. Each bar represents the mean value for three replicates; the error bars show ±standard error of the mean. (B) LPS profiles of two *H. parainfluenzae* isolates and their *lgtF* mutants. 12.5 μl of proteinase K treated cell lysates at OD_260_ = 5 (left panel) or OD_260_ = 1 (right panel) were separated by tricine SDS-PAGE and silver-stained. Labels above each lane are the wild type strain (bold) or *lgtF* mutant clone numbers. LgtF is the glucosyltransferase responsible for the addition of Glc to HepI, and as the Glc is the OAg attachment point, *lgtF* mutants lack this Glc residue and OAg from the LPS. The T3T1 and T3T1.2 profiles contain several protein bands that could not be removed by proteinase K treatment. (C) Association of *H. parainfluenzae* strains T3T1 and 13 and their *lgtF* mutants with human bronchial epithelial cells. Bacteria were incubated in a 96-well plate with a monolayer of 16HBE14 cells. The *y* axes record the number of bacteria that were associated after a 2.5 h incubation as a percentage of the total bacteria in a control well. Each bar represents the mean value for three replicates; the error bars show ±standard error of the mean. Note that both *lgtF* mutants show approximately the same level of association to epithelial cells.

Adherence to host cells is one of the first steps required for the colonisation of the respiratory tract, allowing bacteria to establish themselves in a relatively constant environment whilst avoiding ciliary clearance. For *H. influenzae*, association with epithelial cells is thought to be a prerequisite for invasive disease, as the bacteria must move either through or between the cells of the epithelial barrier to reach the bloodstream. Two OAg-expressing *H. parainfluenzae* strains were compared to *H. influenzae* regarding their ability to invade human bronchial epithelial cells in vitro during a 2.5 h incubation. The percentage of total bacteria that were found inside epithelial cells was 1.9 × 10^−5^% for *H. parainfluenzae* strain 13, 3.3 × 10^−5^% for *H. parainfluenzae* strain 20 and 9.3 × 10^−5^% for *H. influenzae* strain Rd (Fig. 4A). This data is consistent with the hypothesis that of the two species, *H. parainfluenzae* is less able to invade epithelial cells so may be less likely to behave pathogenically.

To examine the role of LPS structure in *H. parainfluenzae* adhesion, association assays were performed using *lgtF* mutants of strains with group 1 or group 2 OAg (Fig. 4B and C). Intriguingly, the effect of ablating the addition of OAg and Glc to HepI of the LPS (i.e. mutating *lgtF*) was strain-dependent. The *H. parainfluenzae* T3T1 *lgtF* mutant, T3T1.2, showed 15 times greater association with epithelial cells than wild type *H. parainfluenzae* T3T1, suggesting that the OAg impairs adherence. For *H. parainfluenzae* strain 13, which has a different OAg structure, the *lgtF* mutant appeared to adhere less than the wild type strain. OAg therefore appears to play opposite roles in adhesion in the two study strains (see Discussion).

## Discussion

LPS is one of the main structural components of the outer membrane in Gram negative bacteria, and in many species the exposed position of OAg makes it a key determinant of interactions with the host. We have demonstrated that all of 18 commensal *H. parainfluenzae* isolates contain a cluster of genes related to polysaccharide synthesis and transport flanked by *glnA* and *pepB*. This provides strong evidence that there is positive selection in *H. parainfluenzae* for the ability to synthesise a polysaccharide structure on its surface via an UndP carrier. Mutation of the UndP sugar transferase gene in several strains confirmed the involvement of the locus in LPS OAg and O-unit synthesis.

We have demonstrated that it is possible to predict some OAg structural details from the genes present in an OAg locus. This has allowed us to generate an overview of O-unit diversity within *H. parainfluenzae* without needing to analyse every structure using chemical methods. Many glycosyltransferase genes found in the OAg loci do not have close homologues in other species in the NCBI database and may have novel donor sugar, acceptor site and/or linkage specificities, making them potentially interesting to glycobiologists attempting to synthesise particular oligosaccharides (Paton et al., 2000) or creating panels of glycans for drug discovery screens. The sugars predicted or proven to form part of these *H. parainfluenzae* OAg structures include Gal*p*, Gal*f*, Gal*p*NAc, Glc*p*NAc, Fuc*p*NAc4N, Neu5Ac, ribitol and deoxytalose, with OAc and PEtn additions.

There was an approximately equal distribution across the *H. parainfluenzae* strains of the two most common OAg synthesis and transport systems. Although many bacterial species comprise a mixture of Wzy-dependent and ABC transporter-dependent OAg serotypes, it is unusual for the two types of OAg loci to map to the same genomic location as they do in *H. parainfluenzae*. This ensures that only one OAg locus is present in each strain. The use of an ABC transporter for heteropolymeric OAg export, as seen in *H. parainfluenzae* strain 13, is rare but not unprecedented (Perepelov et al., 2009; Xu et al., 2010).

Amongst the *H. parainfluenzae* strains tested we found no correlation between the type of OAg locus and the presence of particular *H. influenzae*-like outer core LPS biosynthesis genes that we had previously identified (Young and Hood, 2013) such as *lpsB*, *losB1* and *lic2C*. This emphasises the high degree of genetic exchange that must occur in *H. parainfluenzae*, particularly in the genes required for OAg synthesis. It also increases the likelihood that a strain colonising a new host expresses an LPS structure that has not been encountered by the host's immune system before, thereby extending the average length of colonisation whilst specific antibodies are produced. Indeed, if we combine our genetic data for outer core and OAg-related genes and assume that all strains can express at least one O-unit in vivo, around 16 of the 18 *H. parainfluenzae* strains are predicted to express unique LPS structures.

The diversity of *H. parainfluenzae* OAg structures contrasts strongly with the *H. influenzae* HMG unit, for which the biosynthetic enzymes are also encoded between *glnA* and *pepB* in a vestigial OAg locus; 60% of NTHi strains synthesise the same two non-polymeric tetrasaccharide HMG structures, and the remaining 40% do not have the *hmg* locus (Hood et al., 2004). As the *H. influenzae* outer core structure is highly variable within and between strains due to phase variation, the requirement to display diverse OAgs for immune evasion is likely reduced. Several other mucosal pathogens, including *N. meningitidis*, *Bordetella pertussis* and *Campylobacter jejuni*, also lack OAg on their LPS.

The genomes of one isolate each of *Haemophilus sputorum* and *Haemophilus haemolyticus* have recently been sequenced as part of the NIH Human Microbiome Project (Peterson et al., 2009). The structure and biosynthetic genes of LPS have never been investigated in these species. BLASTP analysis (RY, unpublished data) reveals that both genomes contain an apparent intact polysaccharide biosynthesis locus remarkably similar to the *H. parainfluenzae* strain 13 OAg locus, with homologues of *wzm*, *wzt*, *glf*, *wfdJ*, *wciB*, *rfbB* and *wbaP*. In the *H. sputorum* CCUG13788 locus, genes *13C* to *13E* are replaced by a single glycosyltransferase gene (Fig. S4) and no ligase gene is present. In *H. haemolyticus* HK386 the locus contains a convergent OAg ligase gene (*waaL*) with 78% aa identity to the ligase gene (HI0874) that is found in the same position and orientation in the *H. influenzae* Rd HMG locus (Fig. S4). The results of our earlier BLAST analyses (Tables S1 and S3) suggest that different *H. haemolyticus* strains carry *H. parainfluenzae*-like group 1 and group 2 OAg, respectively. Future electrophoretic and structural LPS analyses of these and other *Haemophilus* species may well reveal that OAg is widespread across the genus, with *H. influenzae* an exception to the rule.

It is clear that other bacteria found in the human respiratory tract are an important source of OAg synthesis genes for *H. parainfluenzae*, although the direction of individual horizontal gene transfer events is difficult to ascertain. Genetic exchange does not appear to be limited to genes from related species or those involved in OAg synthesis, as some of the closest potential homologues found to *H. parainfluenzae* OAg locus genes were capsule and teichoic acid synthesis genes from the Gram positive respiratory tract species *S. pneumoniae* and *Gemella haemolysans*. As *H. parainfluenzae* is reported to colonise the human digestive tract and can be cultured from faecal samples (Palmer, 1981), genetic exchange with species such as *B. fragilis* and *Shigella dysenteriae* may also occur: this was reflected in the BLAST analysis. OAg expression may in fact aid colonisation of the digestive tract by *H. parainfluenzae* as it does for other bacteria (Fowler et al., 2006).

All group 2 and ungrouped *H. parainfluenzae* strains were found to encode a putative OAg ligase outside the OAg locus, between the genes *fba* and *orfH*. This was shown to be required for the synthesis of OAg-containing LPS glycoforms in strain 15. A homologue of this ligase gene is also present next to *fba* in the genome sequences of several other Pasteurellaceae, including species of the genera *Actinobacillus*, *Aggregatibacter*, *Pasteurella* and *Mannheimia*, suggesting that it is either ancestral or has spread through the family very successfully by lateral gene transfer. When Tang and Mintz (2010) interrupted the ligase gene next to *fba* in *A. actinomycetemcomitans*, they observed not only the loss of OAg from the LPS profile but also a shift in the electrophoretic mobility of the adhesin EmaA, which is usually glycosylated with what is thought to be the same ‘OAg’. This glycosylation step was important for the stability of EmaA (Tang et al., 2012). It is possible that the *H. parainfluenzae* homologue is also bifunctional and could glycosylate both LPS and certain outer membrane proteins with identical polysaccharides.

*H. parainfluenzae* may confer some protection against colonisation with more pathogenic species (Van Hoogmoed et al., 2008) but since it is also occasionally implicated in disease, opinion is likely to be divided over whether it would be desirable to retain or reduce colonisation levels. In any case it is important that we understand the potential impact of *H. influenzae* vaccination programmes on the respiratory flora. We found capsule synthesis genes in only one of our 18 *H. parainfluenzae* isolates (strain 18; unpublished data) and the sugar-specific genes were not *H. influenzae*-like, so capsule-based *H. influenzae* vaccines are unlikely to target *H. parainfluenzae*. Regarding potential NTHi vaccines, those based on cell-surface proteins may target both species, whilst those based on the LPS HMG unit (Sundgren et al., 2010) would not impact directly on *H. parainfluenzae* as the O-unit structures produced by this species contain different sugar and linkage combinations to the HMG.

The presence, quantity and structure of polysaccharides on the bacterial cell surface have profound effects upon interactions with host molecules and cells. We demonstrated that in line with findings in other species (Holzer et al., 2009; Merino et al., 1992), OAg contributes to the ability of *H. parainfluenzae* to resist the killing effect of human complement. Long OAg chains can prevent antibodies that recognise conserved membrane proteins from reaching their target (Russo et al., 2009), whilst antibodies binding to the end of the OAg chain can trigger deposition of the complement component C3 away from the bacterial membrane (Goebel et al., 2008). C3 deposition on the outer membrane is required for the formation of the membrane attack complex (MAC) and for targeting bacteria to phagocytes. Although the complement system is associated primarily with serum, complement components are also found in respiratory mucosa, especially during inflammation (Hallstrom and Riesbeck, 2010), so the ability of *H. parainfluenzae* to synthesise OAg is likely to contribute to its high carriage rates.

For *S. enterica* sv. Typhimurium and *Burkholderia cenocepacia*, the presence of particular OAgs on the LPS has been shown to decrease association with host cells (Holzer et al., 2009; Saldias et al., 2009). In cases such as these it is usually postulated that OAg may prevent attachment by masking surface adhesins; this could indeed be true for *H. parainfluenzae* strain T3T1, which attaches more readily to epithelial cells in the absence of OAg. In contrast, one group of OAg structures has been shown to contribute to adhesion. Human epithelial cells secrete a multipurpose β-galactoside-specific lectin called galectin-3 which binds to the epithelial surface and the extracellular matrix (Dumic et al., 2006). LPS structures that contain terminal β-galactoside, such as certain OAg in *Helicobacter pylori* and the outer core in *Neisseria gonorrhoeae*, promote bacterial–host cell adhesion by binding to galectin-3 (Fowler et al., 2006; John et al., 2002). The OAg of *H. parainfluenzae* strain 13 appeared to promote, rather than reduce, adhesion to epithelial cells. This OAg contains a β-galactoside structure; indeed, its O-unit backbone is poly-N-acetyllactosamine, which (albeit with different linkages) was the oligosaccharide that bound galectin-3 most strongly from a panel of 41 potential ligands in a study by Hirabayashi and colleagues (2002). This provides a possible explanation as to the contrasting effects of different *H. parainfluenzae* OAgs on epithelial attachment.

Despite the presence of a potential OAg locus, some of our *H. parainfluenzae* strains did not synthesise any detectable OAg when cultivated under standard laboratory conditions (37 °C on BHI agar). Regulation of the quantity and length of OAgs in response to environmental cues such as temperature, iron concentration or serum concentration can allow bacteria to modify their physical and immunological properties to aid survival (Holzer et al., 2009; Jimenez et al., 2008), and it is likely that under certain in vivo conditions OAg would be upregulated to detectable levels in the *H. parainfluenzae* strains. However, as we have not sequenced all 18 OAg loci we cannot rule out the possibility that some may be non-functional due to mutations. *H. pylori* and *B. fragilis* use DNA repeat slippage and invertible promoters respectively to phase-vary their OAg structures (Cerdeno-Tarraga et al., 2005; Sanabria-Valentin et al., 2007); we found no evidence for either of these sequence features in the five *H. parainfluenzae* OAg loci analysed.

In some species, particular OAg serogroups are associated with increased virulence: for example, most cases of Legionnaire's disease are caused by serogroup 1 strains of *Legionella pneumophila*, which are spread across diverse lineages but share the same OAg genes (Cazalet et al., 2008). The genomes of three clinical *H. parainfluenzae* isolates have recently been sequenced as part of the Human Microbiome Project. Data extracted from NCBI show that all have an OAg gene cluster between *glnA* and *pepB*. In isolates HK262 (from the urogenital tract) and HK2019 (from a facial skin abscess), the locus is 100% identical and encodes proteins typical of a group I *H. parainfluenzae* OAg locus including WcfS, Wzz and WaaL. The OAg locus sequence data for *H. parainfluenzae* ATCC33392, isolated from a septic finger, are incomplete but it also encodes potential homologues of the three aforementioned proteins. This is consistent with, but in no way proves, the hypothesis that only a subset of *H. parainfluenzae* OAg serotypes is capable of causing disease.

## Figures and Tables

**Table 1:** PCR analysis to test the distribution of *H. parainfluenzae* OAg locus genes. Primers were designed to genes in the sequenced *H. parainfluenzae* OAg loci and were used to test for the presence of each gene across the study strains by PCR amplification. *H. parainfluenzae* strain numbers are listed along the top, sorted according to the OAg locus groups which became apparent during the study. The origin of each strain is given below the strain number as either The Gambia (G) or the United Kingdom (UK). PCR results are scored according to the products visible by agarose gel electrophoresis. ‘+’ indicates a PCR in which a product of the expected size was amplified; ‘−’ indicates that no product was detected. Positions are left empty where no PCR was attempted. The *nnaA*–*nnaD* genes are usually found together, so primers were designed only to *nnaB* (NT = not tested). For some genes, a second primer pair was tested on strains that had given negative results. These alternative primers were designed to the strain 19 or strain 20 allele; when a product was obtained only for the second primer pair this is marked as ‘19’ or ‘20’, respectively.

			Group 1 OAg loci							Group 2 OAg loci								Ungrouped			Hybrid	
		Strain	2	19	20	22	24	T3T1	31	8	10	13	14	15	16	18	35	17	30	34	Hy6	Hy11
Gene	Putative function	Primers	UK	G	UK	UK	G	G	G	UK	UK	G	UK	G	UK	G	UK	G	G	G	UK	UK
*H. parainfluenzae T3T1 OAg locus (PARA_)*																						
*02870*	Chain length determinant	P1/P2	−	19	−	19	19	+	19	−	−	−	−	−	−	−	−	−	−	−	−	−
*02860*	NnaD (NeuD)	NT																				
*02850*	NnaB (NeuB)	P3/P4	−	+	−	+	+	+	+	−	−	−	−	−	−	−	−	−	−	−	−	−
*02840*	NnaC (NeuA)	NT																				
*02830*	NnaA (NeuC)	NT																				
*02820*	O-acetyl transferase	P5/P6	−	+	−	−	−	+	−	−	−	−	−	−	−	−	−	−	−	−	−	−
*02810*	OAg flippase	P7/P8	−	+	20	−	−	+	−	−	−	−	−	−	−	−	−	−	−	−	−	−
*02880*	Sialyltransferase	P9/P10	−	+	−	−	−	+	−	−	−	−	−	−	−	−	−	−	−	−	−	−
*02790*	OAg polymerase	P11/P12	−	+	20	−	−	+	−	−	−	−	−	−	−	−	−	−	−	−	−	−
*02780*	Glycosyltransferase	P13/P14	−	+	−	−	+	+	+	−	−	−	−	−	−	−	−	−	−	−		
*02770*	Glycosyltransferase	P15/P16	−	+	20	20	+	+	+	−	−	−	−	−	−	−	−	−	−	−	−	−
*02760*	Aminotransferase	P17/P18	+	+	+	+	+	+	+									−		−		
*02750*	UndP-FucNAc4N P-transferase	P17/P19	+	+	+	+	+	+	+	−	−	−	−	−	−	−	−	−	−	−	−	−
*02740*	UDP-GlcNAc dehydratase	P20/P21	+	+	+	+	+	+	+	−	−	−	−	−	−	−	−	−	−	−	−	−
*02730*	OAg ligase	P22/P23	20	20	20	+	+	+	+	−	−	−	−	−	−	−	−	−	−	−	−	−
*02720*	(dTDP-Glc dehyd.) to *pepB*	P24/P25	+	+	+	+	+	+	+	+	+	+	+	+	+	+	+	+	−	−	−	−

*Strain 13 OAg locus*																						
*glnA* to *13A* (ABC permease)		P26/P27								+	+	+	+	+	+	+	−			−	−	−
*13A*	ABC (permease subunit)	P26/P28	−	−	−	−	−	−	−	+	+	+	+	+	+	+	−	−	−	−	−	−
*13B*	ABC (ATPase subunit)	P29/P30	−	−	−	−	−	−	−	−	+	+	−	+	−	+	−	−	−	−	+	−
*13C*	Glycosyltransferase	P31/P32	−	−	−	−	−	−	−	−	−	+	−	+	−	+	−	−	−	−	−	−
*13D*	Glycosyltransferase	P33/P34	−	−	−	−	−	−	−	−	−	+	−	+	−	+	−	−	−	−	−	−
*13E*	O-acetyl transferase	P35/P36	−	−	−	−	−	−	−	−	−	+	−	+	−	−	−	−	−	−	−	−
*13F*	UDP-Gal*p* mutase	P37/P38	−	−	−	−	−	−	−	+	+	+	+	+	+	+	+	−	−	−	+	+
*13G*	Glycosyltransferase	P39/P40	−	−	−	−	−	−	−	−	−	+	−	−	−	+	+	−	−	−	+	−
*13H*	Glycosyltransferase	P41/P42	−	−	−	−	−	−	−	+	+	+	+	+	+	+	+	−	−	−	+	+
*13I*	UndP-sugar P-transferase	P43/P44	−	−	−	−	−	−	−	+	+	+	+	+	+	+	+	−	−	−	+	+
*13I* to *13J* (dTDP-Glc dehyd.)		P43/P45								+	+	+	+	+	+	+	+				+	+

*17B*	OAg polymerase	P46/P47	−	−	−	−	−	−	−	−	−	−	−	−	−	−	−	+	−	−	−	−
*17C*	OAg flippase	P48/P49	−	−	−	−	−	−	−	−	−	−	−	−	−	−	−	+	−	−	−	−
*17J*	Ribose-5P reductase	P50/P51	−	−	−	−	−	−	−	−	−	−	−	−	−	−	−	+	−	−	−	−
*30B*	Glc1P thymidylyltransferase	P52/P53	−	−	−	−	−	−	−	−	−	−	−	−	−	−	−	−	+	+	−	−
*30E*	ABC (permease subunit)	P54/P55																	+	+		
