# Supplementary material for: Haemophilus parainfluenzae expresses diverse lipopolysaccharide O-antigens using ABC transporter and Wzy polymerase-dependent mechanisms
Source: Int J Med Microbiol. 2013 Dec;303(8):603–17. doi: 10.1016/j.ijmm.2013.08.006 (PMC3989065; doi:10.1016/j.ijmm.2013.08.006)
Supplement: Supplementary file 1 [file mmc1.pdf]

Online Supporting Information

Haemophilus parainfluenzae expresses diverse lipopolysaccharide O-antigens using ABC transporter and Wzy polymerase-dependent mechanisms

Rosanna Young [a, b, *], Brigitte Twelkmeyer [c], Varvara Vitiazeva [c], Peter Power [b], Elke Schweda [c, d] and Derek Hood [b, e]

[a] *Department of Structural and Molecular Biology, University College London, Darwin Building, Gower Street, London, WC1E 6BT, United Kingdom*
[b] *Department of Paediatrics, University of Oxford, United Kingdom (former affiliation)*
[c] *Clinical Research Centre, Karolinska Institutet, Novum, S-141 86 Huddinge, Stockholm, Sweden*
[d] *Division of Chemistry, IFM, Linköping University, SE-581 83 Linköping, Sweden*
[e] *MRC Harwell, Oxfordshire, OX11 0RD, United Kingdom*

* Corresponding author at: Department of Structural and Molecular Biology, University College London, Darwin Building, Gower Street, London, WC1E 6BT, United Kingdom. Tel.: +44 (0)20 7679 2676.
Email address: r.young@ucl.ac.uk (R. Young).

Contents

Table S1	BLASTP analysis of the *H. parainfluenzae* T3T1 O-antigen locus
Table S2	BLASTP analysis of the *H. parainfluenzae* strain 20 O-antigen locus
Table S3	BLASTP analysis of the *H. parainfluenzae* strain 13 O-antigen locus
Table S4	BLASTP analysis of the *H. parainfluenzae* strain 17 O-antigen locus
Table S5	BLASTP analysis of the *H. parainfluenzae* strain 30 O-antigen locus
Table S6	Susceptibility of UndP-sugar phosphotransferase mutant strains to the killing effect of 10% pooled human serum
Table S7	Strain details
Table S8	Primers used for PCR analysis of OAg loci
Table S9	Development of mutant *H. parainfluenzae* strains
Table S10	Plasmid constructs for the interruption of genes in *H. parainfluenzae*
Table S11	Primers used for the construction of recombinant plasmids
Figure S1	LPS profiles following tricine SDS-PAGE using concentrated *H. parainfluenzae* cell lysates
Figure S2	PCR amplification to test for the presence of an OAg ligase gene between the *rfbB* and *pepB* genes
Figure S3	PCR amplification of the strain 15-type OAg ligase
Figure S4	Relationship between the *H. parainfluenzae* strain 13 OAg locus and gene clusters from two other *Haemophilus* species

Table S1. BLASTP analysis of the 16 ORFs in the *H. parainfluenzae* T3T1 O-antigen locus. Searches were carried out on 24 February 2013. The second column lists the putative size of the encoded protein in amino acids (aa), based on the positions of start and stop codons within the locus and on the annotated start codons of homologues. The species containing the three highest scoring BLASTP matches are stated, but only the top match for each genus is included so that the breadth of genera can be shown. For each match the % aa identity is given, with % query coverage in brackets. The putative function of each *H. parainfluenzae* protein is based on the known functions of homologues, domain model searches and in some cases transmembrane domain modelling (see text). For proteins that

may be related to FucNAc4N synthesis or transfer, additional specific BLASTP searches were carried out against species/strains which are known to produce FucNAc4N (shaded in grey).

Gene name	aa	Putative function	Highest scoring BLASTP matches<br>Species	%aa ID (QC)
PARA_02870	377	OAg chain length determinant (Wzz)	*Mannheimia succiniciproducens*	61 (94)
			Haemophilus haemolyticus	51 (100)
			Actinobacillus minor	47 (95)
PARA_02860	209	N-acetylneuraminate synthase / O-acetyltransferase (NnaD)	*Actinobacillus ureae*	74 (99)
			Mannheimia haemolytica	75 (99)
			Simonsiella muelleri	66 (99)
PARA_02850	345	N-acetylneuraminate synthase (NnaB)	*Mannheimia haemolytica*	88 (99)
			Actinobacillus suis	88 (99)
			Simonsiella muelleri	87 (99)
PARA_02840	419	N-acylneuraminate cytidylyltransferase (NnaC)	*Mannheimia haemolytica*	73 (100)
			Actinobacillus suis	69 (100)
			Simonsiella muelleri	60 (100)
PARA_02830	389	UDP-GlcNAc epimerase (NnaA)	*Actinobacillus suis*	71 (98)
			Mannheimia haemolytica	70 (98)
			Simonsiella muelleri	72 (95)
PARA_02820	166	OAc transferase	*Haemophilus haemolyticus*	77 (100)
			Sulfurospirillum barnesii	41 (71)
			Idiomarina baltica	41 (75)
PARA_02810	397	OAg flippase (Wzx)	*Haemophilus haemolyticus*	95 (100)
			Pasteurella dagmatis	57 (99)
			Actinobacillus suis	58 (99)
PARA_02800	300	α-2,3-sialyl transferase (GT family 52)	*Streptococcus agalactiae*	40 (98)
			Mannheimia haemolytica	41 (99)
			Actinobacillus ureae	38 (93)
PARA_02790	357	OAg polymerase (Wzy)	*Escherichia coli*	30 (78)
			Shigella dysenteriae	27 (98)
			Bacteroides stercoris	32 (88)
PARA_02780	323	GT (GTA superfamily)	*Haemophilus haemolyticus*	98 (100)
			Streptococcus thermophilus	39 (99)
			Capnocytophaga sp.	39 (96)
PARA_02770	271	GT (GTA superfamily)	*Haemophilus haemolyticus*	98 (100)
			Bacteroides cellulosilyticus	39 (98)
			Eubacterium rectale	40 (97)
PARA_02760	403	Aminotransferase (FucNAc4N synthesis) (WcfR)	*Haemophilus haemolyticus* M21621	99 (100)
			Bacteroides fragilis NCTC 9343, gene *wcfR*	79 (99)
			Capnocytophaga sp.	76 (100)
			Porphyromonas endodontalis	74 (99)
			Streptococcus pneumoniae R6, gene *aatB*	44 (99)
			Shigella sonnei 53GI, gene *wbgX*	38 (97)
			Plesiomonas shigelloides C27, gene *wbgX*	38 (97)
			Bordetella parapertussis 12822, gene *bplF*	34 (97)
PARA_02750	195	UndP-FucNAc4N phosphotransferase (WcfS)	*Haemophilus haemolyticus* M21621	95 (100)
			Bacteroides fragilis NCTC 9343, gene *wcfS*	72 (100)
			Porphyromonas endodontalis	64 (98)
			Fusobacterium nucleatum	62 (98)
			Shigella sonnei 53GI, gene *wbgY*	54 (98)
			Plesiomonas shigelloides C27, gene *wbgY*	54 (98)
			Bordetella parapertussis 12822, gene *bplG*	56 (98)
PARA_02740	631	UDP-GlcNAc dehydratase (FucNAc4N synthesis) (WbfY)	*Haemophilus haemolyticus* M21621	88 (100)
			Vibrio sinaloensis	59 (99)
			Marinomonas sp.	57 (99)
			Bacteroides fragilis NCTC 9343, gene BF2848	39 (91)
			Streptococcus pneumoniae R6, gene *aatA*	40 (74)
			Shigella sonnei 53GI, gene *wbgZ*	54 (99)
			Plesiomonas shigelloides C27, gene *wbgZ*	54 (99)
			Bordetella parapertussis 12822, gene *bplL*	48 (94)
PARA_02730	414	OAg ligase (WaaL)	*Haemophilus haemolyticus*	61 (95)
			Aggregatibacter aphrophilus	38 (96)
			Mannheimia succiniciproducens	34 (97)
PARA_02720	337	dTDP-glucose 4,6-dehydratase (RfbB)	*Haemophilus haemolyticus*	94 (98)
			Actinobacillus minor	71 (100)
			Neisseria meningitidis	70 (98)

Table S2. BLASTP analysis of the ten ORFs in the *H. parainfluenzae* strain 20 O-antigen locus. See legend for Table S1, except that the intensity of shading for matches to *H. parainfluenzae* T3T1 proteins indicates the degree of similarity. The Genbank protein IDs for 20A-20J are AGO01080 through to AGO01089.

Gene	aa	Putative function	Highest scoring BLASTP matches<br>Species (protein)	%aa ID (QC)
20A	380	OAg chain length determinant (Wzz)	*H. parainfluenzae* T3T1 (PARA_02870)	83 (98)
			Mannheimia succiniciproducens	58 (94)
			Actinobacillus minor	45 (96)
20B	415	OAg flippase (Wzx)	*Bacteroides uniformis*	34 (98)
			Muricauda ruestringensis	36 (84)
			Odoribacter splanchnicus	29 (95)
20C	455	OAg polymerase (Wzy)	*Bacteroides uniformis*	33 (82)
			Sebaldella termitidis	28 (89)
			Leptotrichia goodfellowii	28 (82)
20D	343	Phosphoryl transferase, Stealth protein (WfgC)	*Haemophilus parasuis*	56 (99)
			Nitratifractor salsuginis	50 (100)
			Escherichia coli	37 (99)
20E	270	Glucosyltransferase (WajH)	*Haemophilus haemolyticus*	68 (99)
			(*H. parainfluenzae* T3T1) (PARA_02770)	68 (99)
			Vibrio alginolyticus	39 (99)
			Prevotella buccae	45 (81)
20F	403	Aminotransferase (FucNAc4N synthesis) (WcfR)	*Haemophilus haemolyticus*	99 (100)
			(*H. parainfluenzae* T3T1) (PARA_02760)	99 (100)
			Bacteroides clarus	81 (99)
			Capnocytophaga sp.	76 (100)
20G	195	UndP-FucNAc4N phosphotransferase (WcfS)	*H. parainfluenzae* T3T1 (PARA_02750)	98 (99)
			Bacteroides fragilis	74 (98)
			Alistipes sp.	64 (97)
20H	631	UDP-GlcNAc dehydratase (FucNAc4N synthesis) (WbfY)	*H. parainfluenzae* T3T1 (PARA_02740)	95 (100)
			Vibrio sinaloensis	58 (99)
			Marinomonas sp.	56 (99)
20I	418	OAg ligase (WaaL)	*H. parainfluenzae* T3T1 (PARA_02730)	77 (100)
			Aggregatibacter aphrophilus	37 (97)
			Mannheimia succiniciproducens	33 (97)
20J	337	dTDP-glucose 4,6-dehydratase (RfbB)	*H. parainfluenzae* T3T1 (PARA_02720)	94 (100)
			Actinobacillus minor	70 (100)
			Neisseria meningitidis	70 (98)

Table S3. BLASTP analysis of the ten ORFs in the *H. parainfluenzae* strain 13 O-antigen locus. See legend for Table S1. The Genbank protein IDs for 13A-13J are AGO01052 through to AGO01061.

			**Highest scoring BLASTP matches**	
**Gene**	**aa**	**Putative function**	**Species**	**%aa ID (QC)**
13A	263	ABC transporter, permease subunit (Wzm)	*Actinobacillus pleuropneumoniae*	81 (100)
			Aggregatibacter aphrophilus	81 (98)
			Haemophilus haemolyticus HK386	79 (100)
13B	243	ABC transporter, ATPase subunit (Wzt)	*Haemophilus haemolyticus* HK386	95 (99)
			Actinobacillus pleuropneumoniae	94 (99)
			Aggregatibacter aphrophilus	85 (99)
13C	338	Glycosyltransferase, likely family 2 (WajA)	*Pseudoramibacter alactolyticus*	36 (94)
			Clostridium clostridioforme	37 (98)
			Ruminococcus gnavus	35 (96)
13D	334	Glycosyltransferase, likely family 2 (WajB)	*Clostridium arbusti*	35 (60)
			Myroides injenensis	39 (60)
			Lactobacillus delbrueckii	31 (91)
13E	367	O-acetyltransferase (WajC)	*Erwinia amylovora*	47 (99)
			Actinobacillus succinogenes	48 (99)
			Azospirillum lipoferum	31 (99)
13F	382	UDP-galactopyranose mutase (Glf)	*Haemophilus haemolyticus* HK386	99 (99)
			Actinobacillus pleuropneumoniae	91 (100)
			Aggregatibacter aphrophilus	88 (100)
13G	308	Glycosyltransferase, family 2 (WfdJ)	*Haemophilus haemolyticus* HK386	95 (100)
			Actinobacillus pleuropneumoniae	58 (99)
			Aggregatibacter aphrophilus	53 (98)
13H	256	Glycosyltransferase (WciB)	*Haemophilus haemolyticus* HK386	94 (100)
			Actinobacillus pleuropneumoniae	58 (100)
			Aggregatibacter aphrophilus	53 (99)
13I	471	UndP-sugar phosphotransferase (WbaP)	*Haemophilus haemolyticus* HK386	97 (100)
			Pasteurella bettyae	59 (100)
			Actinobacillus succinogenes	60 (100)
13J	336	dTDP-glucose 4,6-dehydratase (RfbB)	*Haemophilus parainfluenzae* T3T1	94 (99)
			Actinobacillus minor	70 (99)
			Neisseria meningitidis	69 (99)

Table S4. BLASTP analysis of the 14 ORFs in the *H. parainfluenzae* strain 17 O-antigen locus. See legend for Table S1. The capsular serotype (for *S. pneumoniae*) or OAg serotype (for *A. pleuropneumoniae*) of strains which contain the protein are given in brackets to aid prediction of the *H. parainfluenzae* strain 17 OAg structure. The Genbank protein IDs for 17A-17N are AGO01064 through to AGO01077.

Gene	aa	Putative function	Highest scoring BLASTP matches<br>Species (serotype)	%aa ID (QC)
17A	374	OAg chain length determinant (Wzz)	*H. parainfluenzae* T3T1	90 (99)
			Mannheimia succiniciproducens	63 (97)
			Actinobacillus minor	47 (97)
17B	392	OAg polymerase (Wzy)	*Streptococcus macacae*	31 (95)
			Lactobacillus coryniformis	28 (96)
			Eubacterium rectale	27 (97)
17C	476	OAg flippase (Wzx)	*Actinobacillus pleuropneumoniae*	57 (98)
			Pasteurella bettyae	58 (87)
			Catenibacterium mitsuokai	50 (99)
17D	368	UDP-galactopyranose mutase (Glf)	*Streptococcus pneumoniae* (13, 29, 33B, 35B etc.)	87 (99)
			Lactobacillus suebicus	77 (99)
			Granulicatella elegans	78 (99)
			(*H. parainfluenzae* strain 13, gene *13F*)	45 (100)
17E	263	Galactofuranosyl transferase (WciB)	*Granulicatella elegans*	66 (100)
			Streptococcus pneumoniae (17A, 20, 29, 35A, 35B)	67 (100)
			Mannheimia succiniciproducens	57 (99)
			(*H. parainfluenzae* strain 13, gene *13H*)	39 (96)
17F	283	Ribitol phosphotransferase (WefL)	*Streptococcus pneumoniae*	64 (96)
			Blautia hydrogenotrophica	42 (93)
			Capnocytophaga sp.	37 (95)
17G	325	Glycosyltransferase family 2 (WajD)	*Gemella haemolysans*	58 (97)
			Streptococcus oralis	54 (99)
			Granulicatella adiacens	54 (96)
17H	332	Glycosyltransferase family 2 (WajE)	*Streptococcus pneumoniae* (20, 33B, 33F)	50 (96)
			Lachnospiraceae bacterium	41 (98)
			Lactococcus garvieae	39 (99)
17I	235	Ribitol-5P cytidylyltransferase (RblB)	*Streptococcus pneumoniae* (all serotypes)	70 (99)
			Gemella haemolysans	71 (99)
			Granulicatella elegans	59 (99)
17J	340	Ribose-5P reductase (RblA)	*Gemella morbillorum*	64 (100)
			Streptococcus infantis	63 (100)
			Parvimonas micra	59 (100)
17K	321	Glycosyltransferase, likely family 14 (WajF)	*Gemella haemolysans*	50 (97)
			Granulicatella elegans	50 (95)
			Streptococcus mitis	51 (94)
17L	473	UndP-sugar phosphotransferase (WbaP)	*Actinobacillus pleuropneumoniae* (4, 7, 13)	69 (100)
			Pasteurella bettyae	65 (100)
			Haemophilus influenzae (HMG locus)	66 (100)
17M	350	Acyltransferase (WajG)	*Dialister invisus*	44 (98)
			Bacillus mycoides	31 (92)
			Lachnospiraceae bacterium	29 (82)
17N	335	dTDP-glucose 4,6-dehydratase (RfbB)	*Haemophilus haemolyticus*	92 (99)
			H. parainfluenzae T3T1 (PARA_02720)	90 (99)
			Gallibacterium anatis	71 (99)
			Actinobacillus minor	69 (99)

Table S5. BLASTP analysis of the *H. parainfluenzae* strain 30 O-antigen locus. Gene name and length are given in the first two columns. For genes *30C* to *30K,* the highest scoring BLASTP match is from *Aggregatibacter actinomycetemcomitans* (*Aa*) strain D11S-1, with gene order conserved between the two OAg loci (see locus tag numbers). For protein 30A there are higher scoring matches in *Haemophilus paraphrohaemolyticus*, *Actinobacillus minor* and *Neisseria meningitidis* than in *Aa*; for protein 30B there is a closer match in *H. paraphrohaemolyticus* only. For protein 30L there are higher scoring matches from many species than from *Aa*, including *H. influenzae* (58% aa identity). The Genbank protein IDs for 30A-30L are AGO01092 through to AGO01103.

Gene	aa	Putative function	Locus tag of *Aa* match	%aa ID (QC)
30A	354	dTDP-Glc 4,6-dehydratase (RmlB)	D11S_1710	92 (100)
30B	290	Glc-1-phosphate thymidylyltransferase (RmlA)	D11S_1709	94 (99)
30C	294	dTDP-6-deoxy-L-*lyxo*-4-hexulose reductase (dTDP-L-rhamnose synthase; RmlD)	D11S_1708	71 (100)
30D	180	dTDP-6-deoxy-D-*xylo*-4-hexulose-3,5-epimerase (RmlC)	D11S_1707	94 (100)
30E	258	ABC transporter, permease subunit (Wzm)	D11S_1706	90 (99)
30F	247	ABC transporter, ATPase subunit (Wzt)	D11S_1705	83 (99)
30G	633	Rhamnan synthesis protein F (RgpF)	D11S_1704	88 (100)
30H	270	dTDP-6-deoxy-L-*lyxo*-4-hexulose reductase (dTDP-6-deoxy-L-talose synthase; Tll)	D11S_1703	87 (100)
30I	344	Acyl transferase (WajI)	D11S_1702	81 (100)
30J	299	Glycosyltransferase, family 2 (WajJ)	D11S_1701	76 (96)
30K	309	Glycosyltransferase, family 2 (rhamnose?) (WajK)	D11S_1700	79 (98)
30L	472	UndP-sugar phosphotransferase (WbaP)	D11S_1699	47 (86)

Table S6. Susceptibility of UndP-sugar phosphotransferase mutant strains to the killing effect of 10% pooled human serum. The mutant strains lack OAg as a result of interrupting the *wbaP* or *wcfS* gene in the OAg locus. Results are shown as the survival of inoculating bacteria following a 1h incubation in 10% pooled human serum as a percentage of the survival in a 10% decomplemented serum control well.

***H. parainfluenzae* strain**	**19**	**20**	**10**	**13**	**15**	**35**
% survival (wild type strain)	74	92	88	133	99	58
% survival (mutant strain)	1	0	0	0	0	0

Table S7. Strain numbers (as used in the text) and their corresponding full strain names.

Strain number	Strain name
**2**	O/OM30/1/A1
**8**	MO/009/6/S
**10**	O/OMA1/2/B4
**13**	T5T1
**14**	O/OMD4/1/A1
**15**	T6A1
**16**	O/OM41/1/A4
**17**	G/T33T2
**18**	T7B2
**19**	T7A1
**20**	MO/009/1/L
**22**	MO/009/4
**24**	T14A1
**30**	T1A1
**31**	T23A1
**34**	T16B1
**35**	MO/099/61L
**T3T1**	T3T1
**Hy6**	O/OM33/2B3
**Hy11**	MO/099/5/S

Table S8. Primers used for PCR analysis of OAg loci (see Table 1) and amplification of the *H. parainfluenzae* strain 15-like OAg ligase gene (see Fig. S3). Primer sequences are given in the 5' to 3' orientation.

Primer	Sequence
P1	TCAGCCTTCGTTTGTACC
P2	TAATATCCTCTGACAGCC
P3	AATACCATTTCCTGGACG
P4	GCTCCGAAAGCGGAATACC
P5	GCATAAAAGGATATTTCGTAAG
P6	TGAAAGTATTAAAACCAACAAC
P7	CGCCTTACTGATAGGTTCC
P8	AATATGCTAGCAAAGGGG
P9	TGCACAACCCCTTTACAAAC
P10	CACTCCATCGACAACAAAGC
P11	CGAATGCAATACAAACAAGAGC
P12	GTGCGAAATTTTCATTTGAAC
P13	TGGTGGACTGTCTAGTGC
P14	AACTAATACTTCCTTCCC
P15	CAAAACTTGGAGAAATAGACATC
P16	GATGATATAGCTGAAAGAGACC
P17	GTTCTCGAAATTCCAAATAC
P18	ATGCAGCGATTGGCTTAG
P19	TTGAAGATTACCCGCAAGC
P20	CGTGCAGTCTTGCGTTACAT
P21	CCACCAGCTCCTGTTACCAT
P22	GGCGTTTTGGCTAGCATCC
P23	TTCAGATGTAGTGGTACTTTC
P24	GTGCCAAATAAGCCTAATCAC
P25	CCATCACTTGGTTTGATGCT
P26	CCAAGAAAACTCAGCATTAG

P27	ATATTCCAGCGGTAGCAAGC
P28	CCGATTTTATGCATTCGATG
P29	ATTGCGCCATTAATTGAG
P30	CTGCATCGCCCACAGC
P31	TTAGATGAGGCATTAGAACC
P32	CATCAGTATAGAAACATTTCTC
P33	TCTTATGCTAAGAAAGGCTC
P34	GCCTGCTCAAACTCGTTAAG
P35	CCTTCAGGTCCTATTTGGAC
P36	CGATAGAGTAAAATGATAAGG
P37	ACGTGATGCTGAAACAAATG
P38	TTCATTGATTCAGCAAATGG
P39	CAGATTGTCTAGATAAGTTACTCC
P40	AAGTATAAATGCAGAAACATGC
P41	TTATTGGGCTTGGCAAAATG
P42	TCGCCTTCCAAATTAACTAC
P43	TGCATAAAATTACGTGGTG
P44	CCAACGAAAGATACACAGG
P45	GATTGATTTGCCACATAGG
P46	GGAGATAGGCAATTAATAACG
P47	ATAACCTAGAATCAAGCGC
P48	ACGTTTCCTTATGTTTCACG
P49	ACCCCTAAAGATGTAACCAC
P50	ATCCTACACTACTGCGACC
P51	ACTTCCCATTTAAAGATAGTC
P52	GAAAGGTATTATTCTTGCAGG
P53	TTCATCAAATTCCACTACACC
P54	GATAAAGACTCCATTGACAAAC
P55	CAGCCTATCTTCTTACAGGAC
841j	TCGTTACGAATGACTAAAATC
842c	GAAGCAATTAGCTATGGTGC
LIG15B	CCCTCGAATTATGCATATTC
LIG15C	TATGTACCAAGAAACCTTGGC

Table S9. Development of mutant *H. parainfluenzae* strains. The parent strain was transformed using either electroporation (Elec) or the static aerobic method (SA). The donor DNA for transformation was either a plasmid (pRYxx) or chromosomal DNA (chr) from another *H. parainfluenzae* strain as listed. The drug upon which the transformants were selected is given as kanamycin (Kan) or erythromycin (Ery). Note that in this table, *waaL* refers to the OAg ligase gene that is outside the OAg locus.

Mutant name	Parent strain	Genotype	Method	Donor	Drug resistance
10.1	10	*wbaP*	SA	pRY39	Kan
13.1	13	*lgtF*	SA	20.5 chr	Kan
13.2	13	*lgtF*	SA	20.5 chr	Kan
13.7	13	*wbaP*	SA	pRY36	Kan
15.1	15	*lgtF*	SA	19.1 chr	Kan
15.6	15	*wbaP*	SA	10.1 chr	Kan
15.10	15	*waaL*	SA	19.19 chr	Ery
19.1	19	*lgtF*	SA	pRY12	Kan
19.16	19	*wcfS*	SA	pRY41	Kan

19.19	19	*waaL*	SA	pRY46	Ery
20.5	20	*lgtF*	SA	pRY12	Kan
20.18	20	*wcfS*	SA	pRY41	Kan
20.19	20	*wcfS*	SA	pRY41	Kan
T3T1.2	T3T1	*lgtF*	Elec	pRY12	Kan
T3T1.3	T3T1	*lgtF*	Elec	pRY12	Kan
35.9	35	*wbaP*	SA	pRY39	Kan

Table S10. Plasmid constructs for the interruption of genes in *H. parainfluenzae*. In general, a PCR product was amplified using gDNA template from the strain listed in column 3 with the primers given in column 4, and was ligated to the vector in column 5. A drug resistance gene/cassette was then used to interrupt the gene within its 5' end, at the restriction site given in brackets in column 4. Kan^R, kanamycin resistance cassette; ermC, erythromycin resistance gene; USS, uptake signal sequence.

Plasmid	Insert	PCR template strain	Primers (insertion site)	Backbone
**pRY12**	*lgtF* interrupted by Kan^R+USS	T3T1	Hp469-UP and Hp469-DN3 (BglII)	pSCA
**pRY36**	*wbaP* interrupted by Kan^R+USS	13	n/a: HaeIII fragment in clone 5B (Bpu10I)	pBluescript
**pRY39**	*wbaP* partial deletion interrupted by Kan^R+USS	13	HaeIII fragment; deletion by amplification with primers wba13F and wba13R	pBluescript
**pRY41**	*wcfS* partial deletion interrupted by Kan^R+USS	T3T1	272c and 274a; deletion by amplification with primers wba26F and wba26R	pSCA
**pRY46**	*waaL* (*Hp*15-like) interrupted by *ermC*	19	842b and 841j (BsaBI)	pSCA-amp-kan

Table S11. Primers used for the construction of recombinant plasmids. Sequences are given in the 5' to 3' orientation.

Primer	Sequence
Hp469-UP	CATTCGCCACAATGAAAGG
Hp469-DN3	GTACGCCAAACGTCATTACC
272c	GTTCTCGAAATTCCAAATAC
274a	ATGCAGCGATTGGCTTAG
wba13F	gtagtaCAATTGGTCCAATAGTGAAAAAGG
wba13R	gtagtaCAATTGCCATCCCTTGTAATGAT
wba26F	gtagtaCAATTGTCCTCGTCCAGAAGTAAG
wba26R	gtagtaCAATTGCAATCTTAATCCAAATGG
842b	GTCAATTAGGTAACCCTGAAGG
841j	TCGTTACGAATGACTAAAATC

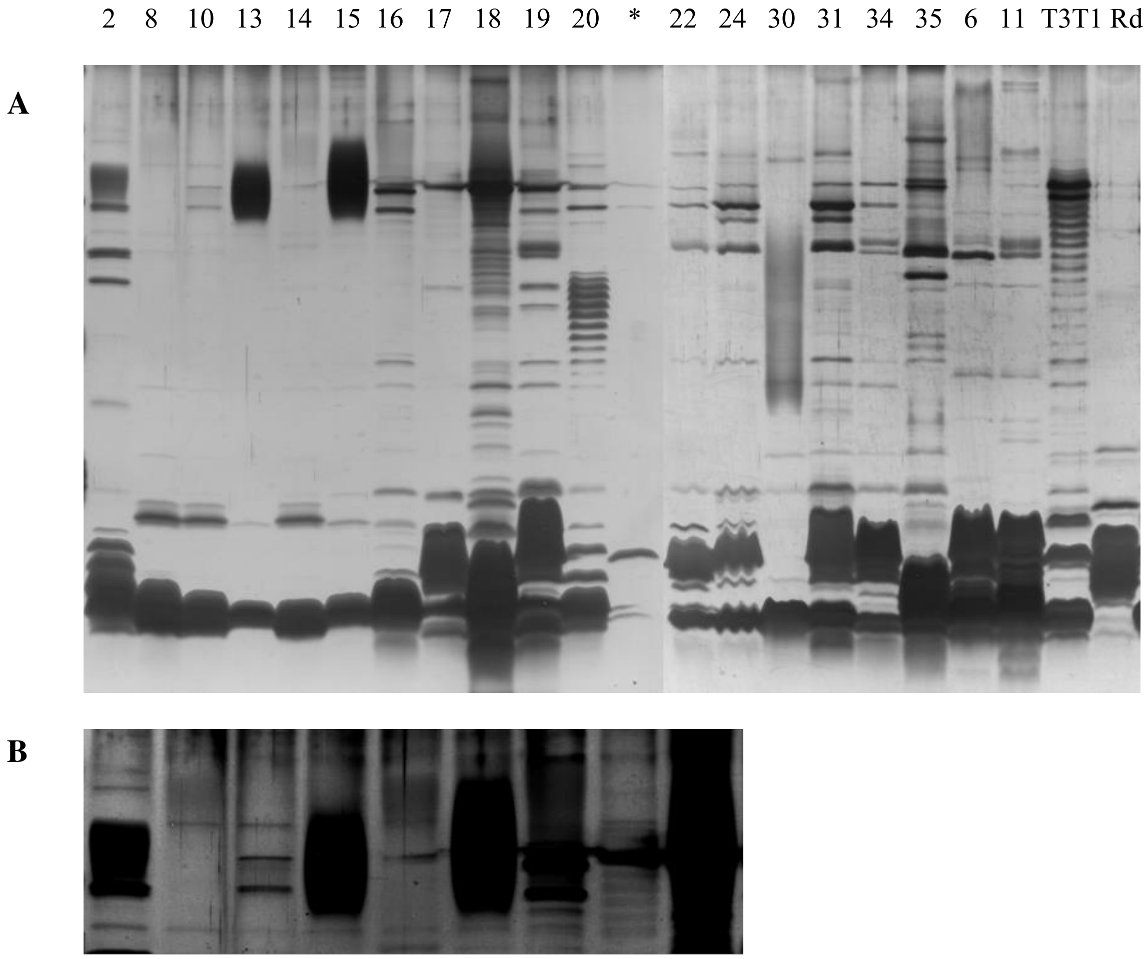

Figure S1. LPS profiles following tricine SDS-PAGE using concentrated *H. parainfluenzae* cell lysates. **(A)** LPS profiles for all 20 strains. Strain numbers are listed above each lane (6 = Hy6, 11 = Hy11). 12.5 µl of lysate at OD_{260} = 10 (rather than the standard OD_{260} = 1) were loaded per lane, in order to visualise any weak OAg that may be present. For strains 13 and 15, lysates at OD_{260} = 5 were used as these strains were already known to produce large quantities of OAg. Strain 24 lysate at the standard concentration was loaded in the lane marked * as a reference. All lysates were treated with proteinase K, but this has not been 100% effective so some protein bands remain. **(B)** Magnified image of the upper region of the first nine lanes of panel (A), with the contrast digitally enhanced so that weak putative OAg can be visualised (e.g. for strains 8 and 17).

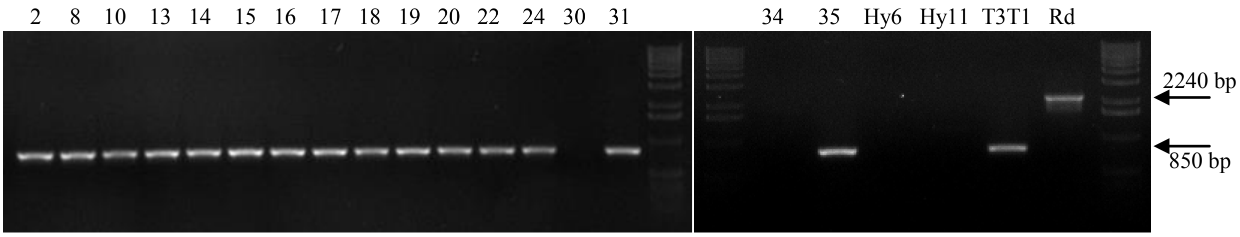

Figure S2. PCR amplification using primers P24 and P25 to test for the presence of an OAg ligase gene between the *rfbB* and *pepB* genes. *H. parainfluenzae* strain numbers are listed above each lane. Unmarked lanes contain 1 kb ladder. T3T1 = strain T3T1 (*rfbB* and *pepB* are consecutive, giving an 850 bp product); Rd = *H. influenzae* strain Rd (the HMG ligase gene is present between *rfbB* and *pepB*, giving a 2.2 kb product). None of the 16 *H. parainfluenzae* strains that give a PCR product appears to have a gene between *rfbB* and *pepB*.

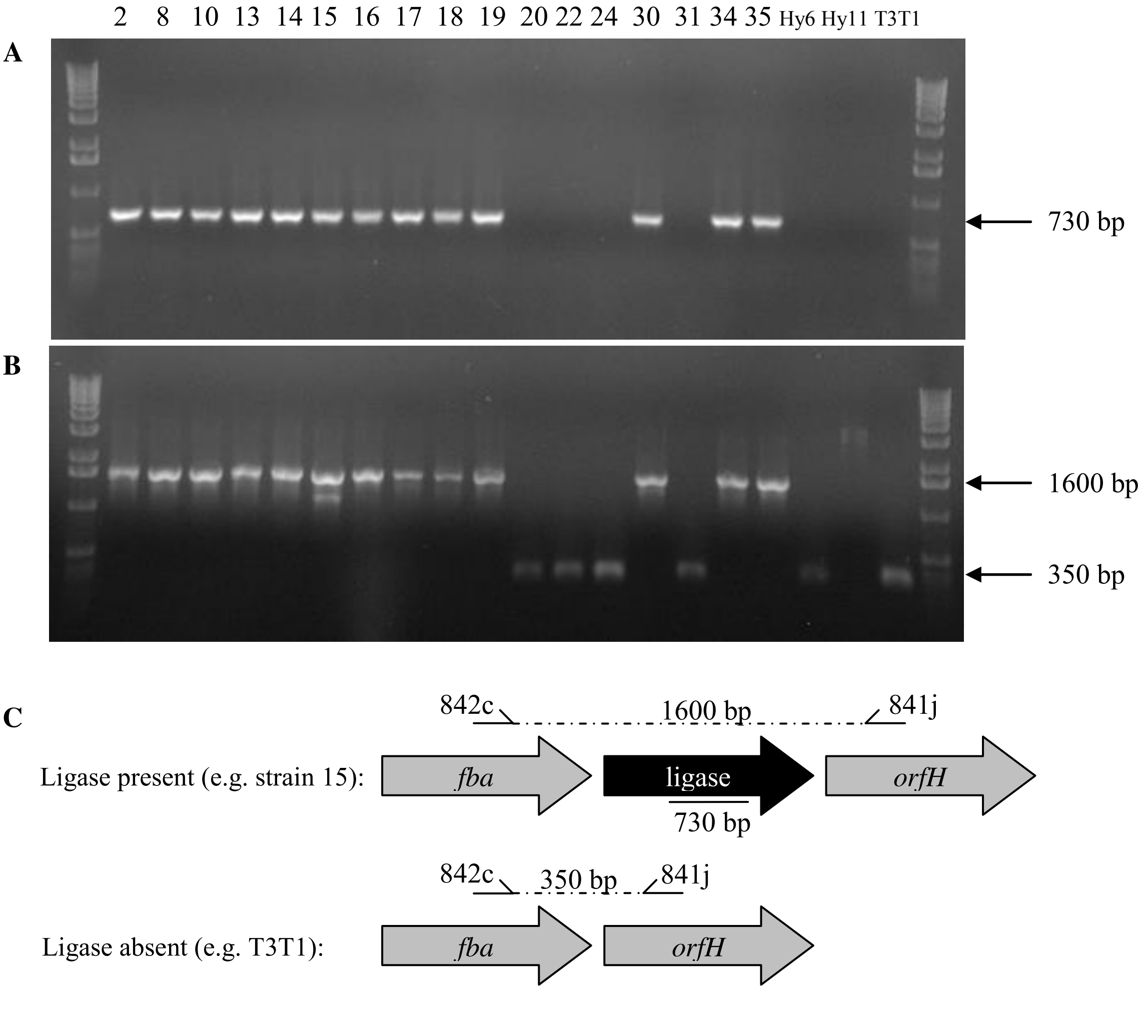

Figure S3. PCR amplification of the strain 15-type OAg ligase. *H. parainfluenzae* strain numbers are listed above each lane. External lanes contain 1 kb ladder. **(A)** Internal primers LIG15B and LIG15C were used to test for the amplification of a 730 bp fragment of the ligase gene from each study strain. 13 of the 20 strains were positive. **(B)** Flanking primers 842c and 841j were used to amplify the ligase locus from each study strain. Products were either 1600 bp (consistent with the presence of a ligase gene at this locus) or 350 bp (consistent with *fba* and *orfH* being contiguous, as in the T3T1 genome). **(C)** Representation of the strain 15 OAg ligase locus and the equivalent region in T3T1. Not drawn to scale.

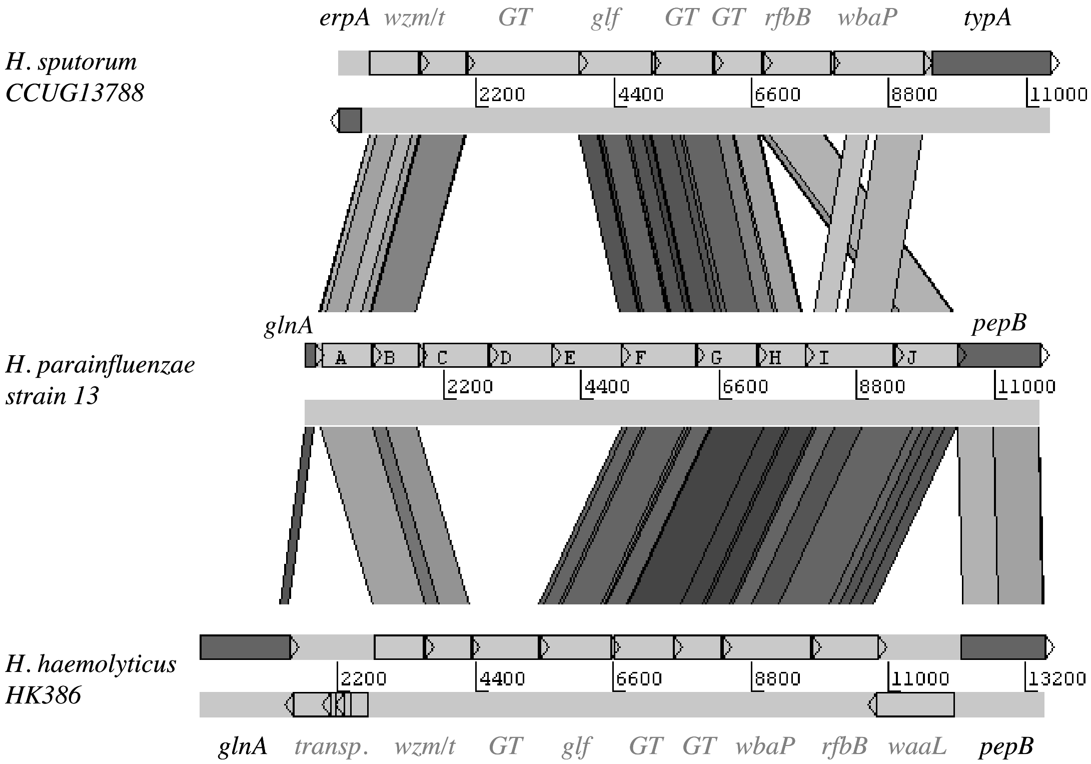

Figure S4. Relationship between the *H. parainfluenzae* strain 13 OAg locus and gene clusters from two other *Haemophilus* species. For each species, ORFs are represented as rectangles with arrowheads indicating the direction of transcription. Gene names (based on similarity to those in strain 13 or Genbank) are given above or below each ORF. GT = glycosyltransferase; transp. = transposon. In *H. haemolyticus* the locus is flanked by *glnA* and *pepB* as it is in *H. parainfluenzae*, whilst in *H. sputorum* it is located between *erpA* and *typA*. TBLASTX analysis between pairs of loci was carried out using DoubleACT v2 software (HPA) and the matching regions that were identified are represented by shaded bands, with the intensity of the shading proportional to the percentage identity of the match. Figure prepared using Artemis and ACT software from the Sanger Institute, UK.
